# Supplementary material for: The serum metabolome serves as a diagnostic biomarker and discriminates patients with melanoma from healthy individuals
Source: Cell Rep Med. 2025 Aug 11;6(8):102283. doi: 10.1016/j.xcrm.2025.102283 (PMC12432359; doi:10.1016/j.xcrm.2025.102283)
Supplement: Document S1. Figures S1–S4 and Tables S1, S3, and S4 [file mmc1.pdf]

Cell Reports Medicine, Volume *6*

Supplemental information

The serum metabolome serves as a diagnostic biomarker and discriminates patients with melanoma from healthy individuals

Yasser Morsy, Barbara Hubeli, Patrick Turko, Marjam Barysch, Julia M. Martínez-Gómez, Nicola Zamboni, Gerhard Rogler, Reinhard Dummer, Mitchell P. Levesque, and Michael Scharl

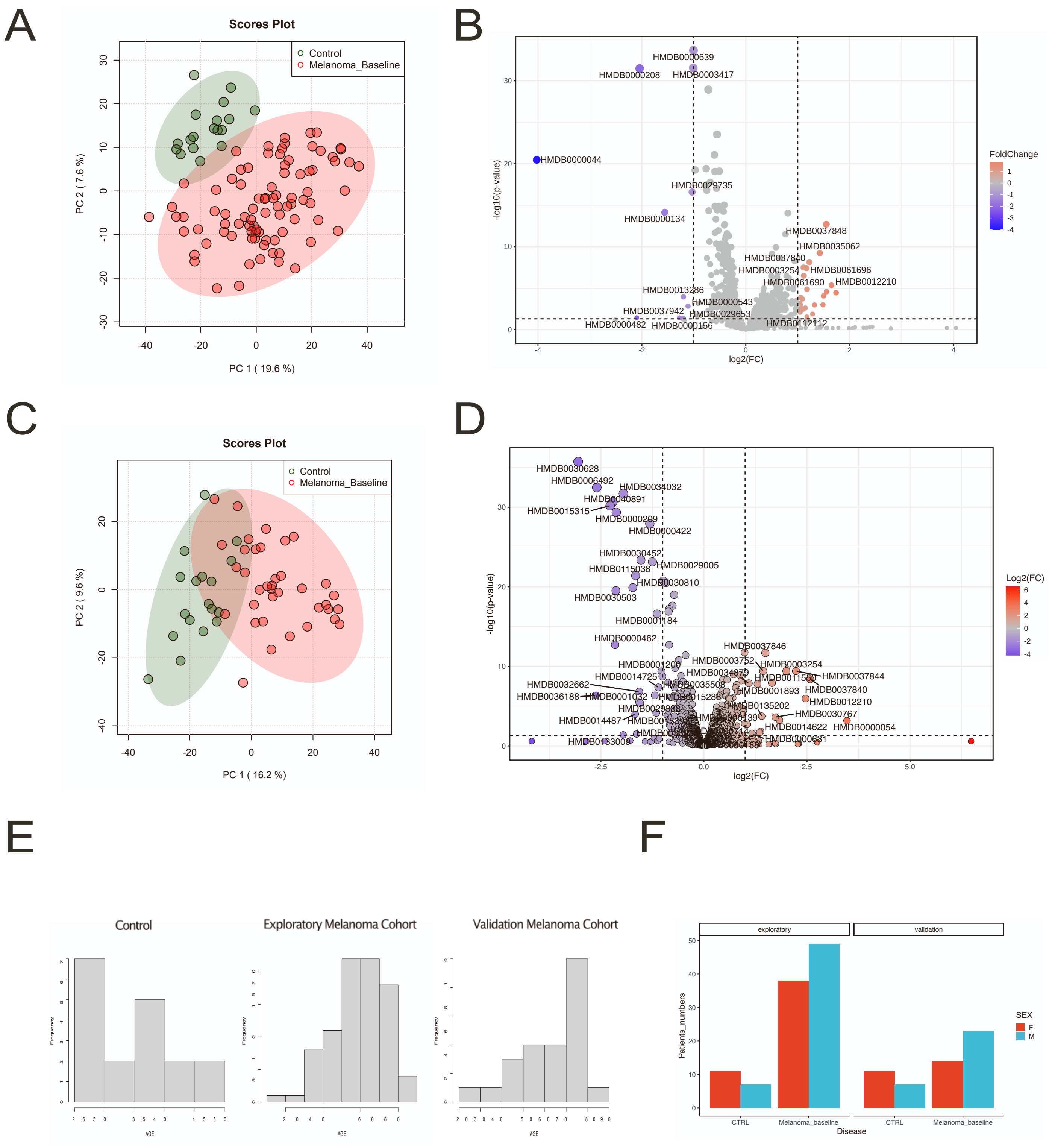

Supplementary Figure 1. Exploratory and validation cohort dimension reduction and volcano plot. Related to Figure 1 and 2 (A) Principle component analysis (PCA) showing a clear separation of the exploratory cohort between 87 melanoma patients and 18 healthy controls based on the metabolites detected. Every dot represents one patient. (B) Volcano plot showing the log2 fold change on the x-axis and -log10 p-value on the y-axis. (C) PCA showing a clear separation of the validation cohort between 37 melanoma patients and 18 healthy controls based on the metabolites detected. Every dot represents one patient. (D) Volcano plot showing the log2 fold change on the x-axis and -log10 p-value on the y-axis. (E) The number of patients categorized by age across the controls and melanoma cohorts. (F) The number of patients categorized by sex across the controls and melanoma cohorts.

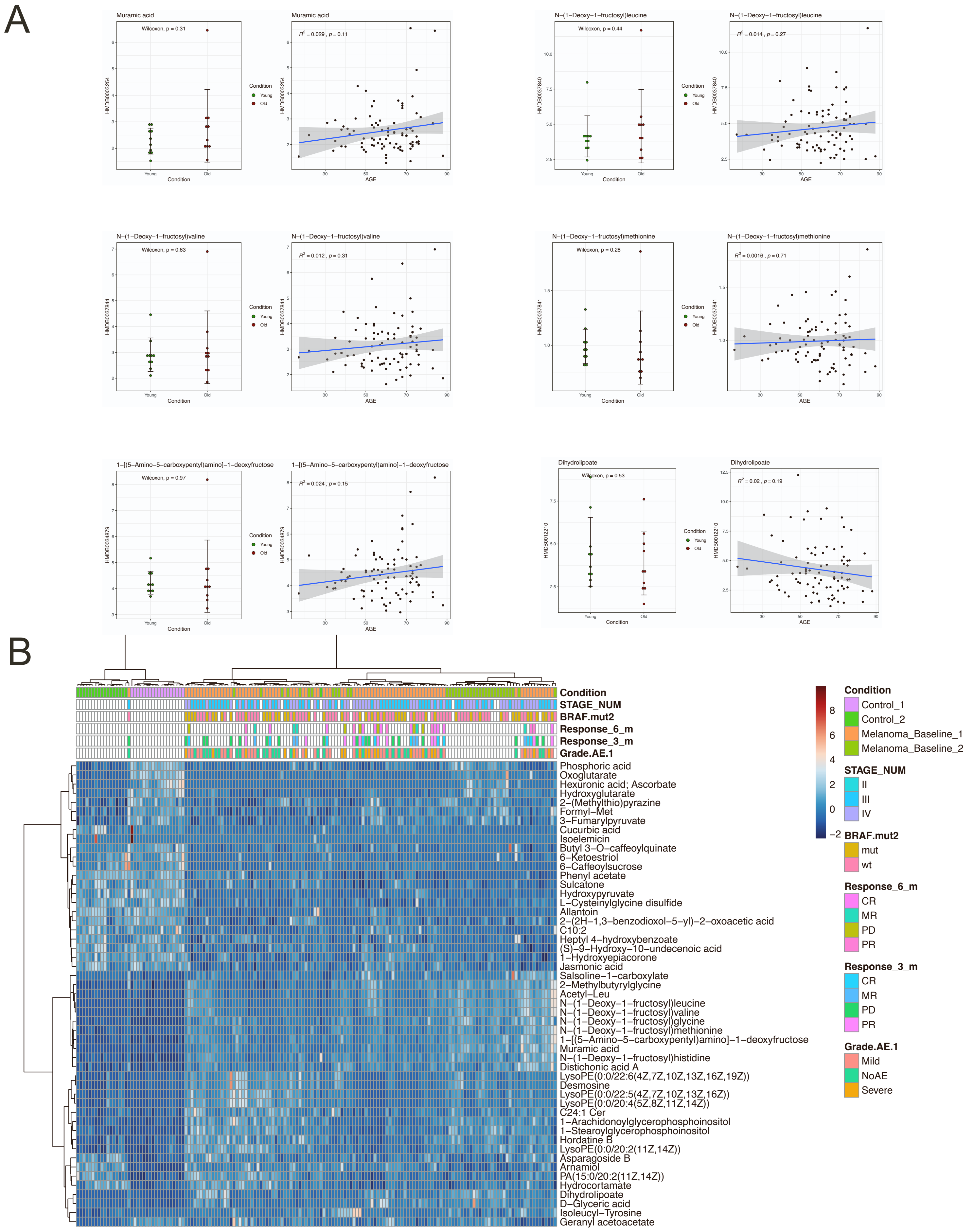

Supplementary Figure 2. Clinical data correlation with biomarkers of patients with stage III or IV melanoma. Related to Figure 3 (A) Ten patients were selected to represent the young patients, and another 10 represented the old patients to check statistics using the Wilcoxon test. For each metabolite, a regression analysis was carried out between the metabolite intensities and patients' ages. Muramic acid, leucine, valine, methionine, deoxyfructose, Dihydrolipoate.Data are represented as mean +/- SD (B) Annotated heatmap showing the absence of clustering based on any clinical data (tumor stage, BRAF mutation, Adverse events, and response after three or six months).

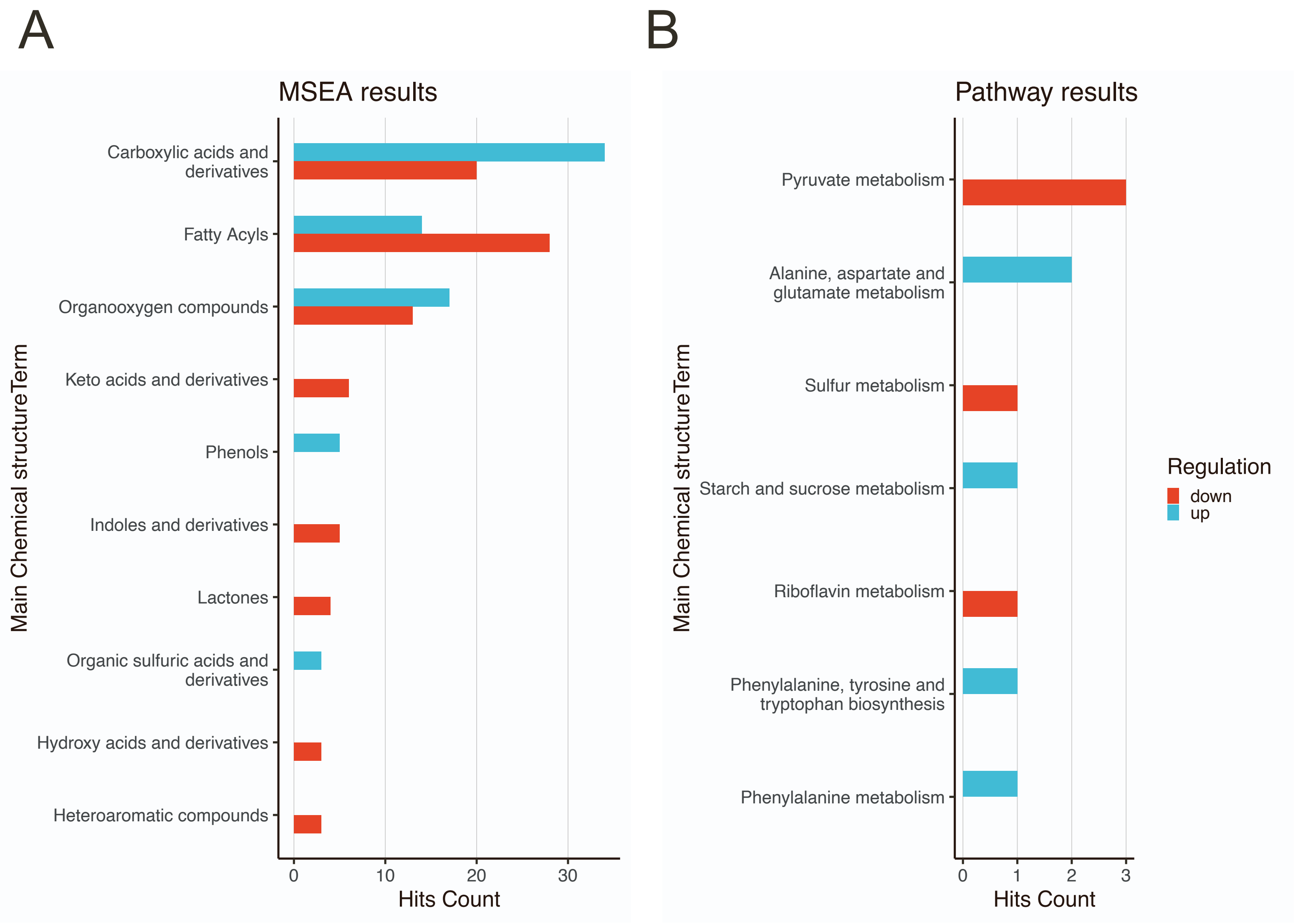

Supplementary Figure 3. Metabolite profiling demonstrates differences in enriched metabolite sets and affects pathways within the serum metabolome between healthy control individuals and patients with early stage melanoma. Related to Figure 4 (A) Bar graph showing top 25 affected metabolite sets after performing metabolite set enrichment analysis (MSEA). (B) Pathway enrichment analysis showing the most affected pathways ranked by the impact of the metabolites in these pathways.

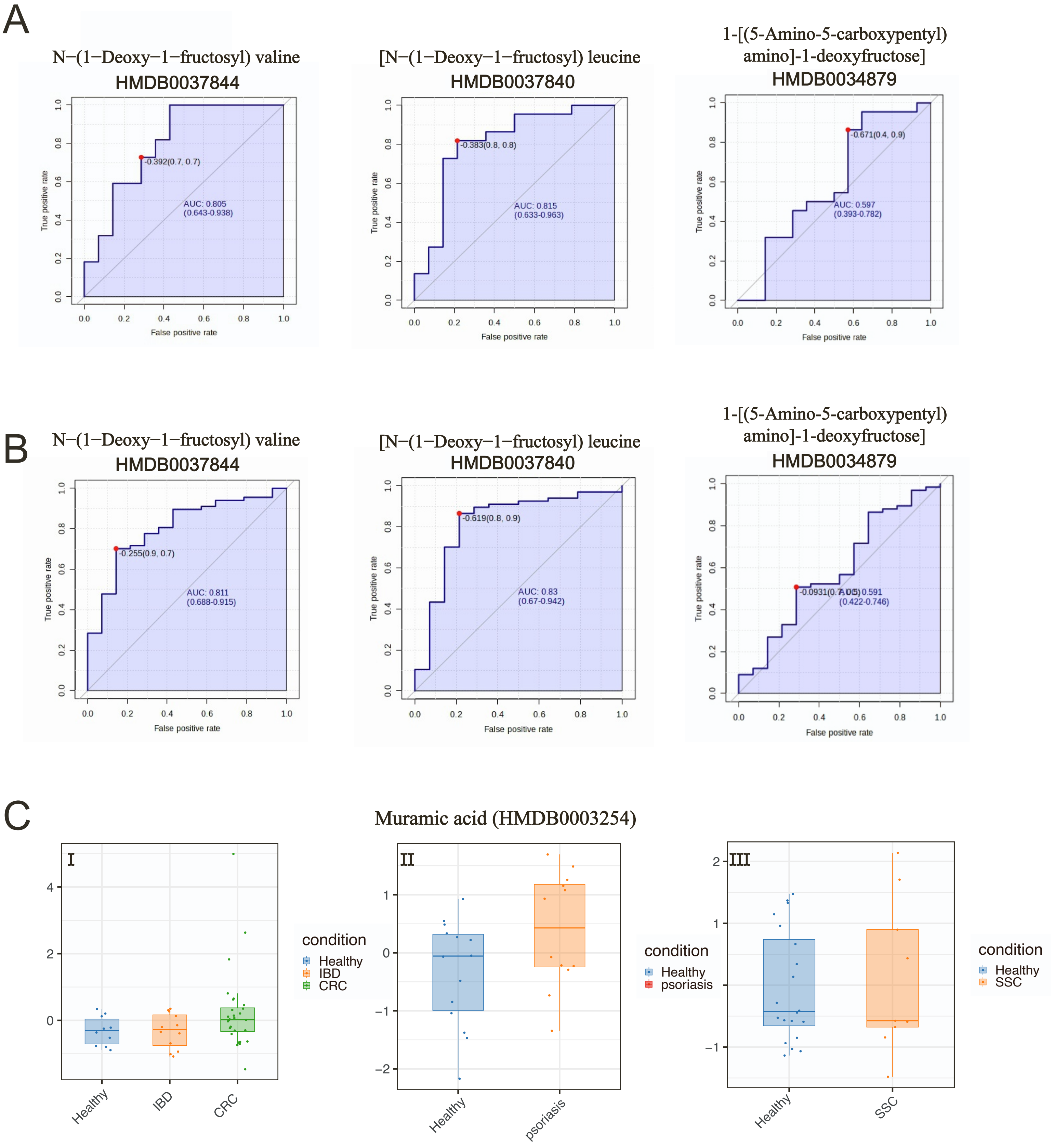

Supplementary Figure 4. Potential serum biomarkers for the diagnosis of patients with melanoma. Related to Figure 4
ROC curves of three top selected metabolites N−(1−Deoxy−1−fructosyl) valine(HMDB0037844), [N−(1−Deoxy−1−fructosyl) leucine (HMDB0037840), 1-[(5-Amino-5-carboxypentyl) amino]-1-deoxyfructose] (HMDB0034879) (A) stage I and (B) stage II patients with melanoma.(C) Centered and scaled levels of Muramic acid (HMDB0003254) across three independent validation cohorts. Comparisons among (I) healthy individuals (n=10) and patients with either Inflammatory Bowel Disease (IBD, n=12) or Colorectal Cancer (CRC, n=28), (II) Healthy individuals (n=14) and patients with Psoriasis (n=12), (III) Healthy individuals (n=20) and patients with Squamous Cell Skin Cancer (SSC, n=9).Data are shown as boxplots with median, interquartile range, and 1.5× IQR whiskers; individual observations are overlaid with jitter.

Supplementary table 1: Patient characteristics of the exploratory and validation cohort Related to Figure 1 and 2

ID_Metabolomics	Disease	SEX	AGE	STAGE_NUM	Cohort
ID1	Melanoma_baseline	F	60	III	exploratory
ID2	Melanoma_baseline	M	57	IV	exploratory
ID3	Melanoma_baseline	F	72	III	exploratory
ID4	Melanoma_baseline	M	54	III	exploratory
ID5	Melanoma_baseline	M	68	IV	exploratory
ID6	Melanoma_baseline	M	72	IV	exploratory
ID7	Melanoma_baseline	M	60	IV	exploratory
ID8	Melanoma_baseline	M	57	III	exploratory
ID9	Melanoma_baseline	F	22	IV	exploratory
ID10	Melanoma_baseline	M	59	III	exploratory
ID11	Melanoma_baseline	M	54	III	exploratory
ID12	Melanoma_baseline	F	65	IV	exploratory
ID13	Melanoma_baseline	M	66	IV	exploratory
ID15	Melanoma_baseline	M	60	IV	exploratory
ID16	Melanoma_baseline	F	58	III	exploratory
ID17	Melanoma_baseline	F	72	III	exploratory
ID18	Melanoma_baseline	F	52	III	exploratory
ID19	Melanoma_baseline	M	38	III	exploratory
ID20	Melanoma_baseline	F	64	IV	exploratory
ID21	Melanoma_baseline	F	40	IV	exploratory
ID22	Melanoma_baseline	M	45	IV	exploratory
ID23	Melanoma_baseline	F	58	III	exploratory
ID24	Melanoma_baseline	M	76	III	exploratory
ID25	Melanoma_baseline	F	38	III	exploratory
ID26	Melanoma_baseline	M	31	II	exploratory
ID27	Melanoma_baseline	F	83	IV	exploratory
ID28	Melanoma_baseline	M	60	III	exploratory
ID29	Melanoma_baseline	F	62	III	exploratory
ID30	Melanoma_baseline	M	68	III	exploratory
ID31	Melanoma_baseline	M	68	IV	exploratory
ID32	Melanoma_baseline	F	70	IV	exploratory
ID33	Melanoma_baseline	F	34	III	exploratory
ID34	Melanoma_baseline	F	35	III	exploratory
ID35	Melanoma_baseline	F	67	III	exploratory
ID36	Melanoma_baseline	M	41	III	exploratory
ID37	Melanoma_baseline	M	58	III	exploratory
ID38	Melanoma_baseline	F	73	III	exploratory
ID39	Melanoma_baseline	F	77	III	exploratory
ID40	Melanoma_baseline	M	67	IV	exploratory
ID41	Melanoma_baseline	M	68	III	exploratory
ID42	Melanoma_baseline	M	48	IV	exploratory
ID43	Melanoma_baseline	M	50	IV	exploratory
ID44	Melanoma_baseline	M	61	III	exploratory
ID45	Melanoma_baseline	M	64	III	exploratory
ID47	Melanoma_baseline	F	53	IV	exploratory
ID49	Melanoma_baseline	F	75	IV	exploratory
ID50	Melanoma_baseline	F	64	III	exploratory
ID51	Melanoma_baseline	F	72	III	exploratory
ID52	Melanoma_baseline	M	49	III	exploratory
ID53	Melanoma_baseline	M	56	IV	exploratory
ID54	Melanoma_baseline	M	56	III	exploratory
ID55	Melanoma_baseline	F	76	III	exploratory
ID56	Melanoma_baseline	M	54	III	exploratory
ID57	Melanoma_baseline	M	70	IV	exploratory
ID58	Melanoma_baseline	M	72	III	exploratory
ID60	Melanoma_baseline	F	49	IV	exploratory
ID61	Melanoma_baseline	M	73	III	exploratory
ID63	Melanoma_baseline	M	84	IV	exploratory
ID64	Melanoma_baseline	M	44	IV	exploratory
ID65	Melanoma_baseline	F	50	III	exploratory
ID66	Melanoma_baseline	M	61	IV	exploratory
ID67	Melanoma_baseline	M	70	III	exploratory
ID68	Melanoma_baseline	F	42	III	exploratory
ID69	Melanoma_baseline	M	53	IV	exploratory
ID70	Melanoma_baseline	F	73	III	exploratory
ID71	Melanoma_baseline	M	72	IV	exploratory

ID72	Melanoma_baseline	F	46	IV	exploratory
ID73	Melanoma_baseline	F	53	III	exploratory
ID74	Melanoma_baseline	F	73	IV	exploratory
ID75	Melanoma_baseline	M	75	IV	exploratory
ID76	Melanoma_baseline	M	64	IV	exploratory
ID77	Melanoma_baseline	M	39	IV	exploratory
ID78	Melanoma_baseline	F	88	IV	exploratory
ID79	Melanoma_baseline	M	55	IV	exploratory
ID80	Melanoma_baseline	F	68	III	exploratory
ID81	Melanoma_baseline	F	53	III	exploratory
ID82	Melanoma_baseline	F	17	IV	exploratory
ID83	Melanoma_baseline	F	68	III	exploratory
ID85	Melanoma_baseline	M	75	III	exploratory
ID86	Melanoma_baseline	M	61	III	exploratory
ID87	Melanoma_baseline	M	54	IV	exploratory
ID88	Melanoma_baseline	M	83	IV	exploratory
ID89	Melanoma_baseline	M	75	III	exploratory
ID90	Melanoma_baseline	F	66	III	exploratory
ID91	Melanoma_baseline	M	35	III	exploratory
ID92	Melanoma_baseline	F	48	IV	exploratory
ID93	Melanoma_baseline	M	73	III	exploratory
ID119	CTRL	F	29	NA	exploratory
ID120	CTRL	F	30	NA	exploratory
ID121	CTRL	F	37	NA	exploratory
ID122	CTRL	M	34	NA	exploratory
ID123	CTRL	M	44	NA	exploratory
ID124	CTRL	M	40	NA	exploratory
ID125	CTRL	M	36	NA	exploratory
ID126	CTRL	F	40	NA	exploratory
ID127	CTRL	F	49	NA	exploratory
ID128	CTRL	F	27	NA	exploratory
ID129	CTRL	M	26	NA	exploratory
ID132	CTRL	F	43	NA	exploratory
ID133	CTRL	F	26	NA	exploratory
ID134	CTRL	M	30	NA	exploratory
ID135	CTRL	F	37	NA	exploratory
ID136	CTRL	M	29	NA	exploratory
ID137	CTRL	F	48	NA	exploratory
ID138	CTRL	F	32	NA	exploratory
ID_199	Melanoma_baseline	M	76	IV	validation
ID_200	Melanoma_baseline	M	78	IV	validation
ID_201	Melanoma_baseline	F	41	III	validation
ID_202	Melanoma_baseline	M	71	IV	validation
ID_203	Melanoma_baseline	F	52	missing	validation
ID_204	Melanoma_baseline	F	83	IV	validation
ID_205	Melanoma_baseline	M	47	III	validation
ID_206	Melanoma_baseline	M	62	IV	validation
ID_207	Melanoma_baseline	M	70	III	validation
ID_208	Melanoma_baseline	F	38	IV	validation
ID_209	Melanoma_baseline	M	52	III	validation
ID_210	Melanoma_baseline	F	54	IV	validation
ID_211	Melanoma_baseline	M	76	III	validation
ID_212	Melanoma_baseline	F	77	III	validation
ID_213	Melanoma_baseline	M	56	III	validation
ID_214	Melanoma_baseline	M	76	IV	validation
ID_215	Melanoma_baseline	M	59	IV	validation
ID_216	Melanoma_baseline	F	59	III	validation
ID_217	Melanoma_baseline	M	79	IV	validation
ID_218	Melanoma_baseline	M	46	IV	validation
ID_219	Melanoma_baseline	M	64	IV	validation
ID_220	Melanoma_baseline	F	77	III	validation
ID_221	Melanoma_baseline	F	80	IV	validation
ID_222	Melanoma_baseline	M	76	III	validation
ID_224	Melanoma_baseline	F	76	III	validation
ID_225	Melanoma_baseline	F	54	III	validation
ID_226	Melanoma_baseline	F	61	IV	validation
ID_227	Melanoma_baseline	M	67	IV	validation
ID_228	Melanoma_baseline	M	65	IV	validation
ID_229	Melanoma_baseline	M	70	IV	validation

ID_231	Melanoma_baseline	M	30	IV	validation
ID_232	Melanoma_baseline	M	78	IV	validation
ID_233	Melanoma_baseline	M	70	IV	validation
ID_234	Melanoma_baseline	F	64	III	validation
ID_235	Melanoma_baseline	M	91	III	validation
ID_236	Melanoma_baseline	M	83	III	validation
ID_237	Melanoma_baseline	F	60	IV	validation
ID119	CTRL	F	29	NA	validation
ID120	CTRL	F	30	NA	validation
ID121	CTRL	F	37	NA	validation
ID122	CTRL	M	34	NA	validation
ID123	CTRL	M	44	NA	validation
ID124	CTRL	M	40	NA	validation
ID125	CTRL	M	36	NA	validation
ID126	CTRL	F	40	NA	validation
ID127	CTRL	F	49	NA	validation
ID128	CTRL	F	27	NA	validation
ID129	CTRL	M	26	NA	validation
ID132	CTRL	F	43	NA	validation
ID133	CTRL	F	26	NA	validation
ID134	CTRL	M	30	NA	validation
ID135	CTRL	F	37	NA	validation
ID136	CTRL	M	29	NA	validation
ID137	CTRL	F	48	NA	validation
ID138	CTRL	F	32	NA	validation

Supplementary table 3: Altered metabolites with fold change above 2 or below 0.5 Related to Figure 1					
ID	Name	FC	log2(FC)	p.ajusted	LOG10(p)
HMDB0011487	LysoPE(0:0/20:4(5Z,8Z,11Z,14Z))	3.3327	1.7367	3.54E-05	4.4507
HMDB0012210	Dihydrolipoate	3.1393	1.6505	4.43E-06	5.3534
HMDB0011494	LysoPE(0:0/22:5(4Z,7Z,10Z,13Z,16Z))	2.9357	1.5537	2.64E-05	4.5785
HMDB0037848	N-(1-Deoxy-1-fructosyl)glycine	2.9247	1.5483	1.99E-13	12.702
HMDB0011485	LysoPE(0:0/20:3(5Z,8Z,11Z))	2.8213	1.4964	8.51E-05	4.0702
HMDB0011496	LysoPE(0:0/22:6(4Z,7Z,10Z,13Z,16Z,19Z))	2.7988	1.4848	0.001028	2.988
HMDB0035062	Arnamiol	2.6853	1.4251	5.65E-10	9.2479
HMDB0000572	Desmosine	2.5008	1.3224	0.0010576	2.9757
HMDB0029369	Neosaxitoxin	2.4364	1.2847	0.01334	1.8748
HMDB0037840	N-(1-Deoxy-1-fructosyl)leucine	2.3352	1.2235	7.27E-09	8.1383
HMDB0000139	D-Glyceric acid	2.2644	1.1791	1.35E-05	4.8682
HMDB0112112	FAHFA(18:0/9-O-18:0)	2.2584	1.1753	0.028059	1.5519
HMDB0061696	1-Stearoylglycerophosphoinositol	2.2451	1.1668	3.90E-08	7.4091
HMDB0011479	LysoPE(0:0/18:3(9Z,12Z,15Z))	2.2128	1.1459	0.0024223	2.6158
HMDB0061690	1-Arachidonoylglycerophosphoinositol	2.1683	1.1166	3.15E-07	6.5014
HMDB0003254	Muramic acid	2.1589	1.1103	3.20E-08	7.4943
HMDB0009215	PE(18:4(6Z,9Z,12Z,15Z)/P-18:1(11Z))	2.1576	1.1095	0.0034943	2.4566
HMDB0011473	LysoPE(0:0/16:0)	2.127	1.0888	0.0002018	3.6951
HMDB0002925	8,11,14-Eicosatrienoic acid	2.0816	1.0577	0.0001331	3.8759
HMDB0001976	C22:5	2.0732	1.0519	0.0020444	2.6894
HMDB0008984	PE(16:1(9Z)/P-18:1(11Z))	2.0712	1.0504	0.0009435	3.0253
HMDB0029854	1,2-Epoxy-1,2,7,7'',8,8'',11'',12''-octahydro-psi,psi-carotene	2.0687	1.0487	0.008059	2.0937
HMDB0002226	C22:4	2.0511	1.0364	0.0006779	3.1688
HMDB0000044	Hexuronic acid; Ascorbate	0.061426	-4.025	3.44E-21	20.464
HMDB0000482	Octanoic acid	0.23347	-2.0987	0.036361	1.4394
HMDB0000208	Oxoglutarate	0.24249	-2.044	3.35E-32	31.475
HMDB0000134	Fumarate	0.33903	-1.5605	7.08E-15	14.15
HMDB0037942	(1xi,3xi)-1,2,3,4-Tetrahydro-1-methyl-beta-carboline-3-carboxylic acid	0.4118	-1.28	0.037748	1.4231
HMDB0000156	Malate	0.4256	-1.2324	0.04383	1.3582
HMDB0013286	N-Undecanoylglycine	0.43474	-1.2018	0.0001087	3.9636
HMDB0029653	1-(1-Methoxy-1-methylethyl)-4-methylbenzene	0.4372	-1.1936	0.045555	1.3415
HMDB0000543	Cumate	0.46245	-1.1126	0.0014671	2.8335
HMDB0029735	5-Ethynyl-5''-(1-propynyl)-2,2''-bithiophene	0.48866	-1.0331	2.54E-17	16.596
HMDB0003417	Cys	0.49635	-1.0106	2.88E-32	31.541
HMDB0000639	Galactaric acid	0.4973	-1.0078	2.16E-34	33.665

Supplementary table 4: Patient characteristics of the second validation cohort Related to Figure 4				
ID_Metabolomics	Disease	SEX	AGE	STAGE_NUM
ES_1	Melanoma_baseline	M	56	I
ES_2	Melanoma_baseline	M	69	I
ES_3	Melanoma_baseline	M	31	II
ES_4	Melanoma_baseline	M	71	II
ES_5	Melanoma_baseline	M	76	I
ES_6	Melanoma_baseline	M	83	II
ES_7	Melanoma_baseline	F	74	I
ES_8	Melanoma_baseline	F	63	II
ES_9	Melanoma_baseline	F	83	I
ES_10	Melanoma_baseline	F	56	II
ES_11	Melanoma_baseline	M	70	I
ES_12	Melanoma_baseline	M	67	I
ES_13	Melanoma_baseline	M	74	II
ES_14	Melanoma_baseline	M	35	I
ES_15	Melanoma_baseline	M	73	I
ES_16	Melanoma_baseline	F	46	II
ES_17	Melanoma_baseline	M	65	I
ES_18	Melanoma_baseline	M	50	II
ES_19	Melanoma_baseline	M	82	II
ES_20	Melanoma_baseline	F	32	II
ES_21	Melanoma_baseline	M	83	II
ES_22	Melanoma_baseline	F	85	II
ES_23	Melanoma_baseline	M	71	II
ES_24	Melanoma_baseline	F	56	II
ES_25	Melanoma_baseline	M	74	II
ES_26	Melanoma_baseline	M	83	I
ES_27	Melanoma_baseline	M	81	I
ES_28	Melanoma_baseline	M	58	II
ES_29	Melanoma_baseline	F	87	II
ES_30	Melanoma_baseline	M	55	I
ES_31	Melanoma_baseline	M	58	II
ES_32	Melanoma_baseline	F	62	II
ES_33	Melanoma_baseline	M	71	II
ES_34	Melanoma_baseline	M	74	II
ES_35	Melanoma_baseline	M	48	I
ES_36	Melanoma_baseline	F	81	I
ES_37	Melanoma_baseline	F	58	II
ES_38	Melanoma_baseline	F	64	II
ES_39	Melanoma_baseline	M	75	II
ES_40	Melanoma_baseline	M	85	II
ES_41	Melanoma_baseline	F	44	II
ES_42	Melanoma_baseline	M	59	II
ES_43	Melanoma_baseline	M	45	II
ES_44	Melanoma_baseline	M	64	II
ES_45	Melanoma_baseline	M	70	II
ES_46	Melanoma_baseline	F	67	II
ES_47	Melanoma_baseline	F	86	II
ES_48	Melanoma_baseline	M	75	II
ES_49	Melanoma_baseline	M	76	II

ES_50	Melanoma_baseline	F	90	II
ES_51	Melanoma_baseline	F	55	II
ES_52	Melanoma_baseline	M	72	II
ES_53	Melanoma_baseline	F	46	I
ES_54	Melanoma_baseline	F	58	I
ES_55	Melanoma_baseline	F	84	II
ES_56	Melanoma_baseline	F	52	II
ES_57	Melanoma_baseline	M	57	II
ES_58	Melanoma_baseline	F	31	I
ES_59	Melanoma_baseline	F	53	I
ES_60	Melanoma_baseline	M	74	II
ES_61	Melanoma_baseline	F	64	II
ES_62	Melanoma_baseline	M	81	II
ES_63	Melanoma_baseline	M	76	II
ES_64	Melanoma_baseline	F	77	I
ES_65	Melanoma_baseline	F	51	II
ES_66	Melanoma_baseline	F	79	II
ES_67	Melanoma_baseline	M	67	II
ES_68	Melanoma_baseline	M	86	II
ES_69	Melanoma_baseline	M	76	II
ES_70	Melanoma_baseline	F	47	II
ES_71	Melanoma_baseline	M	80	II
ES_72	Melanoma_baseline	M	70	II
ES_73	Melanoma_baseline	M	45	II
ES_74	Melanoma_baseline	M	55	II
ES_75	Melanoma_baseline	F	70	II
ES_76	Melanoma_baseline	M	63	II
ES_77	Melanoma_baseline	M	74	II
ES_78	Melanoma_baseline	M	61	II
ES_79	Melanoma_baseline	M	72	II
ES_80	Melanoma_baseline	F	55	II
ES_81	Melanoma_baseline	M	67	I
ES_82	Melanoma_baseline	M	61	II
ES_83	Melanoma_baseline	F	83	II
ES_84	Melanoma_baseline	F	81	II
ES_85	Melanoma_baseline	M	67	I
ES_86	Melanoma_baseline	M	62	II
ES_87	Melanoma_baseline	F	60	II
ES_88	Melanoma_baseline	M	68	II
ES_89	Melanoma_baseline	M	75	II
C_ES_1	Control	M	51	NA
C_ES_2	Control	M	55	NA
C_ES_3	Control	M	36	NA
C_ES_4	Control	M	40	NA
C_ES_5	Control	M	49	NA
C_ES_6	Control	M	67	NA
C_ES_7	Control	M	75	NA
C_ES_8	Control	M	34	NA
C_ES_9	Control	F	42	NA
C_ES_10	Control	F	32	NA
C_ES_11	Control	F	46	NA
C_ES_12	Control	F	25	NA
C_ES_13	Control	M	32	NA
C_ES_14	Control	F	44	NA
