# Supplementary material for: The serum metabolome serves as a diagnostic biomarker and discriminates patients with melanoma from healthy individuals
Source: Cell Rep Med. 2025 Aug 11;6(8):102283. doi: 10.1016/j.xcrm.2025.102283 (PMC12432359; doi:10.1016/j.xcrm.2025.102283)
Supplement: Document S2. Article plus supplemental information [file mmc3.pdf]

Cell Reports
Medicine

Report

The serum metabolome serves as a diagnostic biomarker and discriminates patients with melanoma from healthy individuals

Graphical abstract

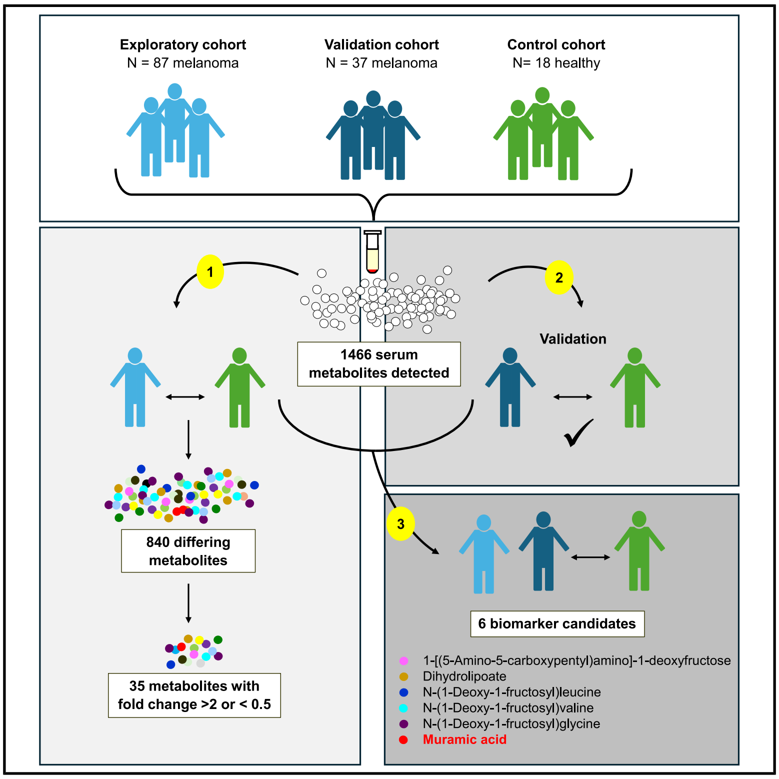

Highlights

- The serum metabolome differs between patients with melanoma and healthy individuals
- Six serum metabolites can predict the diagnosis of melanoma
- Muramic acid is a particularly powerful diagnostic biomarker across melanoma stages

Authors

Yasser Morsy, Barbara Hubeli, Patrick Turko, ..., Reinhard Dummer, Mitchell P. Levesque, Michael Scharl

Correspondence

michael.scharl@usz.ch

In brief

Morsy et al. present an untargeted metabolomics analysis of patients with melanoma, revealing a distinct metabolome composition compared to healthy control individuals. Six specific serum metabolites can accurately diagnose melanoma in a clinical setting. Particularly, the lead biomarker muramic acid represents a very promising accuracy across all melanoma stages.

Morsy et al., 2025, Cell Reports Medicine 6, 102283
August 19, 2025 © 2025 The Author(s). Published by Elsevier Inc.
https://doi.org/10.1016/j.xcrm.2025.102283

CellPress

Cell Reports Medicine

CellPress
OPEN ACCESS

Report

The serum metabolome serves as a diagnostic biomarker and discriminates patients with melanoma from healthy individuals

Yasser Morsy,[1] Barbara Hubeli,[1] Patrick Turko,[2] Marjam Barysch,[2] Julia M. Martínez-Gómez,[2] Nicola Zamboni,[3] Gerhard Rogler,[1] Reinhard Dummer,[2,4] Mitchell P. Levesque,[2,4,5] and Michael Scharl[1,4,5,6,*]
[1]Department of Gastroenterology and Hepatology, University Hospital Zurich, University of Zurich, 8091 Zurich, Switzerland
[2]Department of Dermatology, University Hospital Zurich, University of Zurich, 8091 Zurich, Switzerland
[3]Institute of Molecular Systems Biology, Federal Institute of Technology Zurich, 8093 Zurich, Switzerland
[4]Comprehensive Cancer Center Zurich, University Hospital Zurich, University of Zurich, 8091 Zurich, Switzerland
[5]These authors contributed equally
[6]Lead contact
*Correspondence: michael.scharl@usz.ch
https://doi.org/10.1016/j.xcrm.2025.102283

SUMMARY

Melanoma is a deadly cancer with increasing incidence and mortality rates, and biomarkers for diagnosis are urgently needed. The impact of the microbiome, genetic factors, and immunologic markers on disease outcomes is described, but a comprehensive serum metabolome profiling is missing. The serum metabolome of patients with melanoma might be valuable to identify potential biomarkers. We present an untargeted metabolomics analysis in an exploratory cohort (87 patients with melanoma), an independent validation cohort (37 additional patients with melanoma featuring late-stage tumors), and 18 healthy control individuals, revealing striking differences. We identify and validate six serum metabolites that can predict the diagnosis of melanoma with an area under the curve (AUC) >0.9544 in advanced-stage melanoma. The AUC of our lead biomarker, muramic acid, is 0.964, 0.908, and 0.9936 in patients with stage I (n = 22), stage II (n = 67), and advanced melanoma (n = 86), respectively. In summary, we identify potentially very powerful diagnostic biomarkers for clinical practice.

INTRODUCTION

Melanoma represents one of the most deadly cancers worldwide, and not only incidence rates but also mortality rates keep increasing.[1–3] The further rise in incidence seems to be caused primarily by the increased exposure to UV irradiation, which is one of the biggest risk factors for melanoma.[4] Notably, melanoma is not only frequent in elder patients but has also become one of the most common cancers in young adults.[5] Projected estimates suggest that by 2040, the global burden of melanoma might reach about 500,000 newly diagnosed cases and about 100,000 melanoma-related deaths per year.[6] Such numbers highlight the urgency of highly accurate and easily measurable biomarkers in daily clinical practice, since early detection and treatment of melanoma are critical to improve the prognosis for patients.[3] Currently available biomarkers are particularly related to the correct histopathologic diagnosis as well as to staging, prognosis, and monitoring of therapeutic efficacy of specific therapies. Among those biomarkers are approaches based on the detection of circulating tumor cells, serum nucleic acid levels, gene and protein expression levels and patterns, as well as genetic variations.[7] However, reliable biomarkers for the diagnosis of malignant melanoma are not available. The currently used serum levels of lactate dehydrogenase or S-100 protein are particularly useful only for monitoring disease course or recurrence but not for diagnostic purposes.[8,9]

The microbiome has recently gained enormous attention in cancer research, and some studies suggested that distinct microbiome patterns might serve as biomarkers for the prediction of response to cancer immunotherapies[10–12] or the onset of immune-related side effects.[8] However, to date, neither distinct microbiome compositions nor specific bacterial strains play a role as biomarkers in the clinical standard of care, and the observed associations are mostly not suited to predict the diagnosis of melanoma in patients. However, the observed associations between particular microbiomes of patients with melanoma and therapy outcomes are strong, and it thus seems plausible that the microbiome functionality might represent a useful tool for biomarker detection.

One of the most important functional aspects of the microbiome is its metabolic activity. Therefore, the metabolome might not only be important for disease and therapy outcomes in patients with melanoma but might also serve as a potent biomarker in such cases. Notably, current knowledge about the serum metabolome in patients with melanoma is scarce. Most studies addressing the role of the metabolome in melanoma focused on

 © 2025 The Author(s). Published by Elsevier Inc.
This is an open access article under the CC BY license (http://creativecommons.org/licenses/by/4.0/).

melanoma cells or melanoma tissue[13–17] or even on the fecal metabolome.[18–20] Only one study provided data on the serum metabolome in patients with melanoma.[21] Although these authors applied targeted metabolomics, they, nevertheless, described a significant difference between patients with melanoma and healthy individuals, when analyzing 181 metabolites in the serum of 26 patients with melanoma stage III or IV.[21] Furthermore, in this small and unvalidated cohort, the authors also identified distinct metabolites that might serve as biomarkers for diagnosis of advanced melanoma.[21]

Here, by performing untargeted serum metabolomics analysis, we demonstrate a significant difference in the composition of the serum metabolome between patients with melanoma and healthy individuals and provide a complete overview of the serum metabolome in patients with melanoma. In addition, by conducting our analysis in both an exploratory and an independent validation cohort, we were able to identify six serum metabolites as clinically relevant biomarkers for the diagnosis of advanced melanoma, while our lead candidate even exhibited a very strong biomarker performance in early-stage melanoma.

RESULTS

Untargeted serum metabolome analysis reveals a distinct metabolome composition in patients with melanoma compared to healthy individuals

To determine the complete serum metabolome composition of patients with melanoma, we prospectively collected serum samples from an exploratory cohort of 87 patients with melanoma as well as from an independent validation cohort of 37 additional patients with melanoma featuring late-stage tumors. Samples were collected at baseline just before the initiation of cancer immunotherapy. In addition, we studied serum samples from 18 healthy control individuals in the same age range as a control group. Patient characteristics are shown in Table S1. The principal component analysis (PCA) of the exploratory cohort (87 patients with melanoma) and 18 control individuals demonstrated a clear separation between the two patient groups (Figure S1A). Similarly, the partial least squares discriminant analysis (PLS-DA), which was used to visualize the variance between samples, demonstrated a clear separation between samples from healthy controls and patients with melanoma (Figure 1A). This distinct separation indicates that the serum metabolome of patients with melanoma strongly differs from that of healthy individuals, suggesting that the serum metabolome can potentially identify patients with melanoma and thus be useful for diagnostic purposes. This distinct separation was further corroborated by the heatmap of the 50 most prominent metabolites, which visually presents the hierarchical clustering using the Euclidean method for distance calculation and Ward's linkage for clustering (Figure 1B). While some overlap is observed due to the broader set of metabolites, which further separate the patients into two different melanoma subgroups, this analysis reinforces the overall distinction in metabolic profiles associated with melanoma. Out of the 1,466 detected metabolites, 840 significantly differed between the two groups (Table S2). Furthermore, 35 metabolites showed fold changes above 2 or below 0.5, indicating a substantially different abundance in patients with melanoma and healthy controls. Among them, 23 metabolites showed higher serum levels in patients with melanoma, including dihydrolipoate, N-(1-deoxy-1-fructosyl)glycine, and muramic acid, while 12 metabolites showed lower serum levels compared to healthy controls, including ascorbate, octanoic acid, oxoglutarate, and fumarate (Figure S1B; Table S3).

Functional serum metabolome analysis reveals an enrichment of lipids, fatty acyls, and organic acids in patients with melanoma

We next aimed to identify the metabolite groups that are most prominently regulated in the serum of patients with melanoma when compared to healthy control individuals. For this purpose, the metabolite set enrichment analysis (MSEA) was performed against the "main" dataset, and a generalized linear model was used during the quantitative enrichment analysis. The 840 significantly altered metabolites were also used in MSEA and pathway analysis, highlighting key metabolic pathways affected in patients with melanoma. We found a distinct enrichment of specific classes of metabolites, including lipids and lipid-like molecules, fatty acyls, organic acids, as well as glycerophospholipids and sterol lipids, in the serum of patients with melanoma (Figure 1C). Performing a pathway enrichment analysis, we detected that the most important metabolic pathways enriched in the serum of patients with melanoma were ubiquinone and other terpenoid-quinone biosynthesis, alpha-linolenic acid metabolism, and amino acid metabolism (Figure 1D).

The validation cohort confirms distinct serum metabolome profiles for patients with melanoma and healthy controls

After demonstrating the pronounced difference between the serum metabolome of patients with melanoma and that of healthy controls, we next aimed to confirm our findings in an independent validation cohort consisting of 37 additional patients with melanoma. Again, serum was collected from patients with melanoma at baseline before the initial start of cancer immunotherapy. Further, we again measured the same serum samples from our control cohort consisting of 18 healthy individuals. In this validation cohort, we were able to corroborate our previous findings from the exploratory cohort (Figure 1). We again detected a clear separation between the serum samples from healthy controls and patients with malignant melanoma, in both the PCA and PLS-DA (Figures 2A and S1C), as well as in the heatmap presenting the 50 most prominent metabolites (Figure 2B). However, in this smaller validation cohort, we did not detect the previously observed further separation of patients with melanoma into two distinct subgroups, albeit at least a trend toward such discrimination was noticeable. The volcano plot further demonstrated significantly up- and downregulated metabolites (Figure S1D). The functional metabolome analysis of our validation cohort fully confirmed the results of the MSEA of the exploratory cohorts. In our validation cohort, we again detected a clear enrichment of lipids and lipid-like molecules, fatty acyls, and organic acids (Figure 2C). However, pathway enrichment analysis revealed different subtypes of amino acid metabolisms as the most affected metabolic pathways in the serum of patients with melanoma (Figure 2D).

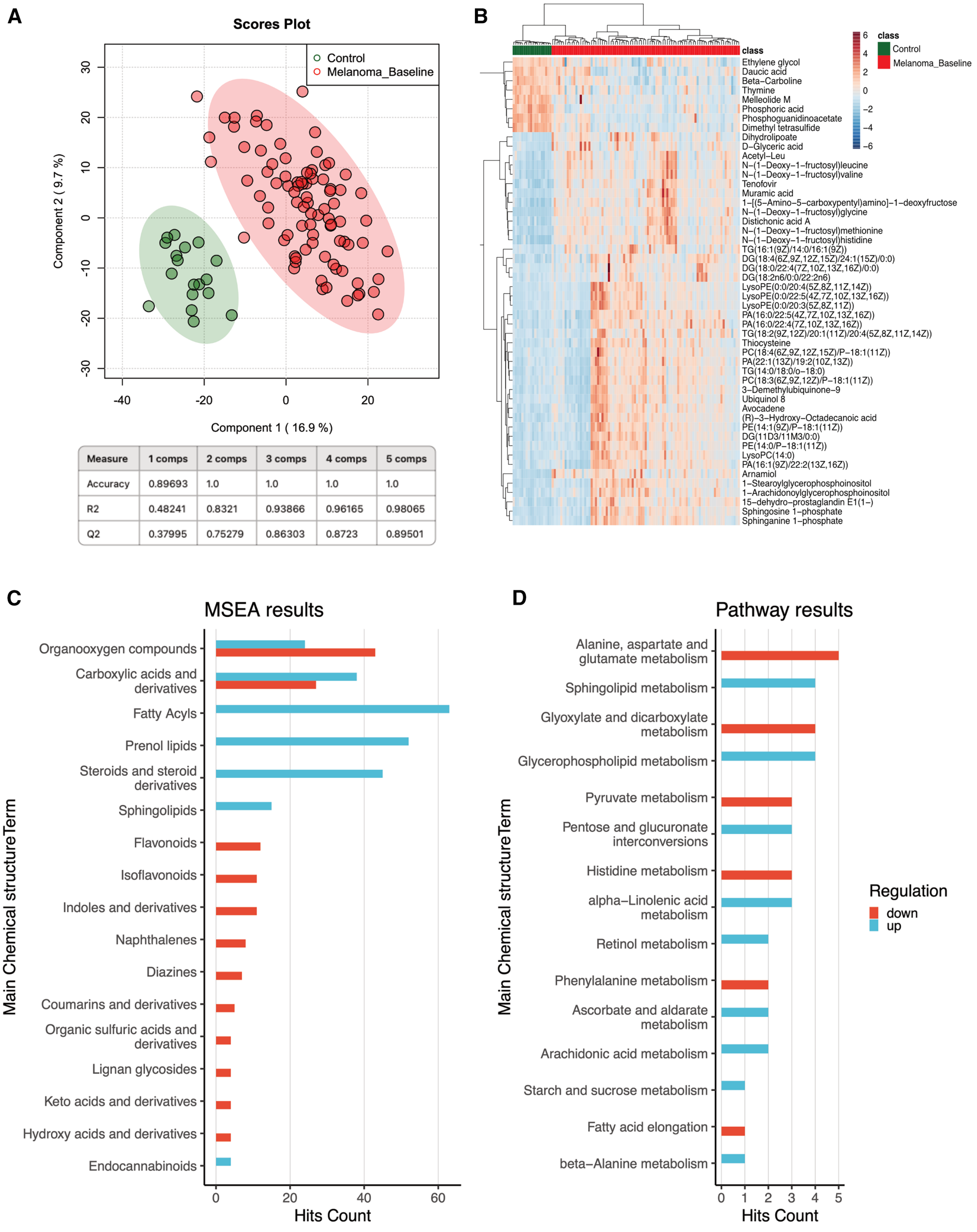

Measure	1 comps	2 comps	3 comps	4 comps	5 comps
Accuracy	0.89693	1.0	1.0	1.0	1.0
R2	0.48241	0.8321	0.93866	0.96165	0.98065
Q2	0.37995	0.75279	0.86303	0.8723	0.89501

Figure 1. Multivariate analysis and metabolite profiling demonstrate striking differences in the serum metabolome between healthy control individuals and patients with melanoma

(A) Partial least squares discriminant analysis (PLS-DA) showing a clear separation between 87 patients with melanoma and 18 healthy controls based on the 1,466 metabolites detected.

(B) Heatmap showing the top 50 significant metabolite changes across melanoma and control individuals.

(C) Bar graph showing top 25 affected metabolite sets after performing metabolite set enrichment analysis (MSEA).

(D) Pathway enrichment analysis showing the most affected pathways ranked by the impact of the metabolites in these pathways.

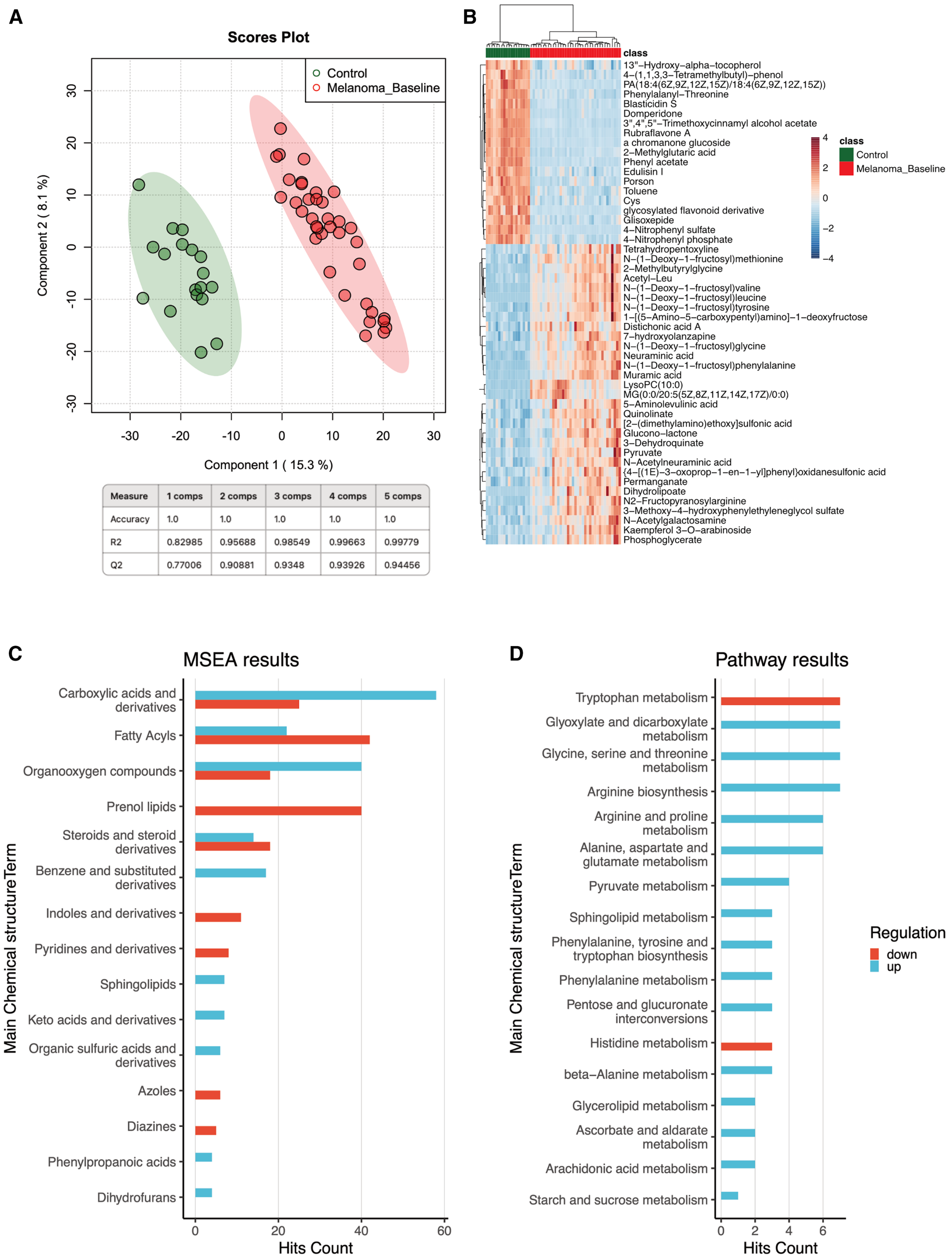

Measure	1 comps	2 comps	3 comps	4 comps	5 comps
Accuracy	1.0	1.0	1.0	1.0	1.0
R2	0.82985	0.95688	0.98549	0.99663	0.99779
Q2	0.77006	0.90881	0.9348	0.93926	0.94456

Figure 2. Serum metabolome data from a validation cohort consisting of 37 patients with melanoma confirm the difference between healthy control individuals and patients with melanoma

(A) Partial least squares discriminant analysis (PLS-DA) showing a clear separation between 37 patients with melanoma and 18 healthy controls based on the 1,466 metabolites detected.

(B) Heatmap showing the top 50 significant metabolite changes across melanoma and control individuals.

(C) Bar graph showing the top 25 affected metabolite sets after performing metabolite set enrichment analysis (MSEA).

(D) Pathway enrichment analysis showing the most affected pathways ranked by the impact of the metabolites in these pathways.

CellPress
OPEN ACCESS

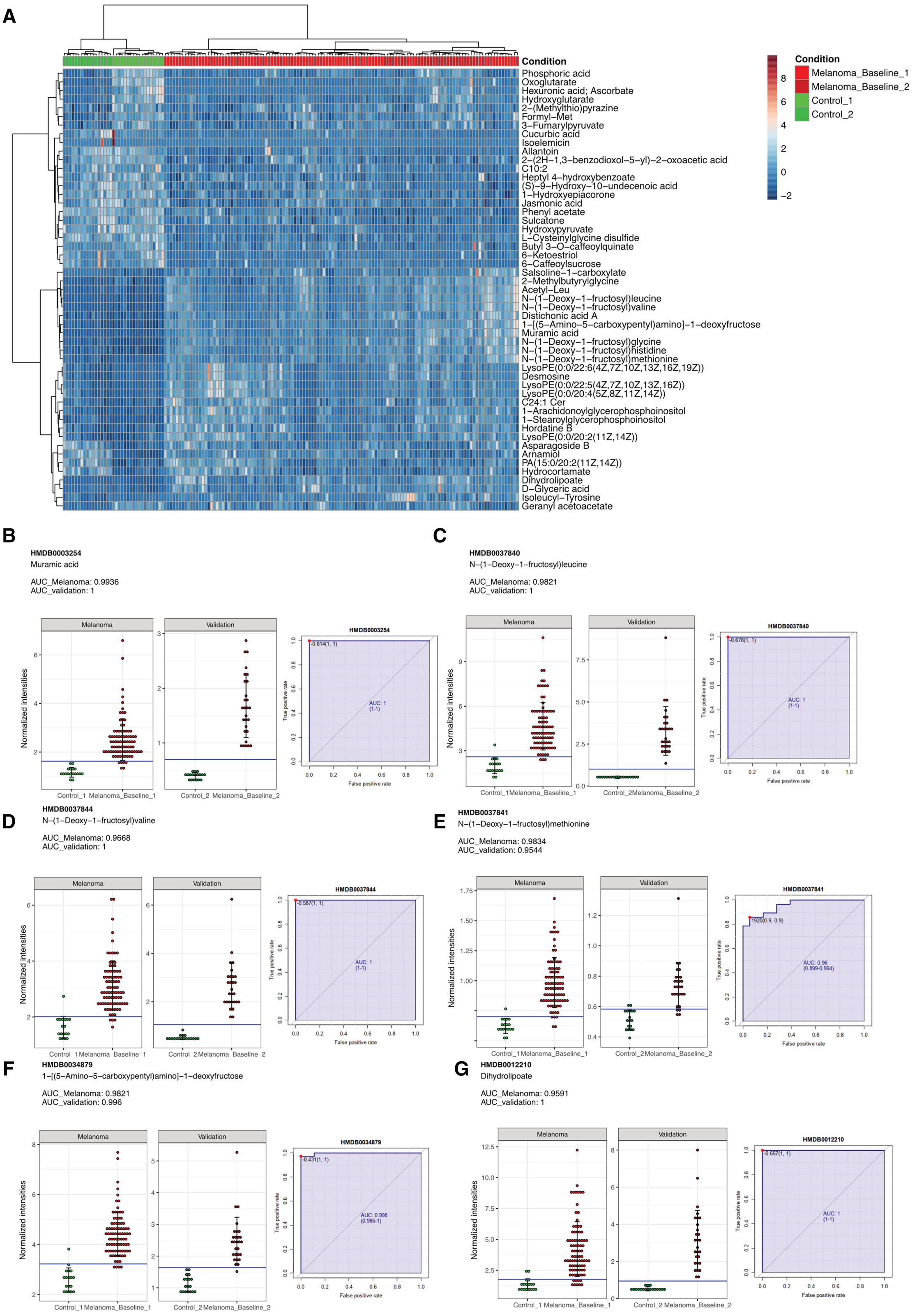

(legend on next page)

Specific serum metabolites may serve as clinically relevant biomarkers for the diagnosis of patients with melanoma

After validating our observations from the exploratory cohort in the validation cohort, we next compared the serum metabolome data of both cohorts and aimed to identify specific metabolites that could serve as clinically relevant biomarkers for the diagnosis of melanoma. Although the cohorts were analyzed separately, we generated a combined heatmap illustrating the top 50 most prominent metabolites identified in both cohorts to visualize their collective discriminatory potential, which confirmed the clear separation between samples from the patients with melanoma and the samples of the healthy controls (Figure 3A). By performing receiver operating characteristic (ROC) curve and area under the curve (AUC) analysis, we evaluated the performance of the top metabolites as biomarkers, and only metabolites with an AUC greater than 0.95 in each cohort were considered as strong candidates for biomarker selection. With this approach, we detected six serum metabolites that might serve as powerful biomarkers for the diagnosis of melanoma in clinical practice. Particularly, we found that six metabolites with false discovery rate less than 0.05, namely 1-[(5-amino-5-carboxypentyl)amino]-1-deoxyfructose (fold change [FC]: 0.80 in melanoma and 1.06 in validation), dihydrolipoate (FC: 1.69 and 2.43), N-(1-deoxy-1-fructosyl)leucine (FC: 1.26 and 2.58), N-(1-deoxy-1-fructosyl)valine (FC: 0.97 and 2.23), N-(1-deoxy-1-fructosyl) glycine (FC: 1.58 and 0.87), and muramic acid (FC: 1.15 and 1.98), exhibited an AUC of at least 0.9544 in both the exploratory and the validation cohort (Figures 3B–3G). The best performance was detected for the bacterial wall component muramic acid, which featured AUCs of 0.9936 and 1.0 for the diagnosis of melanoma in the exploratory cohort and in the validation cohort, respectively. Notably, all six biomarkers were strongly upregulated in the serum of patients with melanoma when compared to healthy controls in both the exploratory as well as the validation cohort.

The selected biomarkers exhibited a notable AUC cutoff of 0.9, indicating their high discriminatory capacity in distinguishing melanoma cases. These biomarkers were overlapped across both the exploratory and validation cohorts, affirming their consistent identification and reliability in differentiating melanoma cases. Moreover, quantitative analysis showcased uniform intensity measurement for these biomarkers across various samples, emphasizing their stability and potential as reliable indicators for melanoma detection or prognosis.

The tested biomarkers were not affected by the age or gender of the respective patients (Figures S1E, S1F, and S2A). Also, there was no obvious contribution of clinical characteristics, such as tumor stage, BRAF mutation status, onset of immune-related adverse events, or therapy response to the clustering (Figure S2B).

Expanding diagnostic biomarkers: Serum metabolites validate melanoma across all stages

We broadened our study to validate the biomarker aspect of the identified molecules also in patients with early-stage melanoma. Thus, we included 22 patients with stage 1 melanoma, 67 patients with stage 2 melanoma, and 14 control subjects (Table S4). Our findings indicate that the metabolomics profile of patients with early-stage melanoma again differs from that of control individuals, as demonstrated by both PLS-DA and hierarchical clustering of altered metabolites (Figures 4A and 4B). Subsequently, we analyzed the enriched metabolites and discovered that they were comparable to those in later stages, with carboxylic acids, fatty acyls, and organooxygen compounds identified as the most highly enriched metabolite groups (Figure S3A). Furthermore, amino acid metabolism emerged as the primary pathway category enriched in our pathway analysis, suggesting that this metabolic process plays a role in disease progression from its early stages (Figure S3B). Consequently, leucine and valine derivatives may serve, among others, as effective functional biomarkers for the early diagnosis of melanoma (Figures S4A and S4B). Finally, we assessed the AUC of our lead biomarker candidate, namely muramic acid, and found that it features an AUC of 0.964 in patients with stage I melanoma as well as of 0.908 in patients with stage II melanoma. In addition, we evaluated its level across three independent cohorts of patients with inflammatory bowel disease (IBD), colorectal cancer (CRC), psoriasis, and squamous cell skin cancer. Of note, we did not observe a significant alteration of muramic acid levels in any of those three cohorts when compared to the levels detected in the serum of healthy individuals (Figure S4C).

DISCUSSION

We uncovered potentially clinically relevant biomarkers for the diagnosis of melanoma using untargeted metabolomics to generate serum metabolite profiles of two independent cohorts of patients with melanoma.

Overall, we uncovered significant alterations in lipid metabolism, organic acid pathways, and amino acid synthesis metabolic pathways between patients with melanoma and healthy controls. These pathways correspond nicely with the known pathophysiological mechanisms involved in the development of melanoma,[22,23] and they were consistently modified across both cohorts. Alterations in lipid metabolism were a prominent feature in our analysis, as evidenced by the enrichment of lipid-related metabolites. This is in line with previously reported data showing that melanoma cells reprogram their lipid metabolism to support rapid proliferation and metastasis.[24] Moreover, increased lipid synthesis and uptake, as observed in our cohorts, are hallmarks of cancer cell metabolism, maintaining membrane fluidity and energy storage.[25,26]

Figure 3. Serum metabolomics analysis identifies potential serum biomarkers for the diagnosis of patients with melanoma
(A) Heatmap of the top 50 identified biomarkers with an AUC greater than 0.95 across both the exploratory and validation cohorts.
(B–G) Intensity levels of the top 6 selected metabolites (muramic acid, N-(1-deoxy-1-fructosyl)leucine, N-(1-deoxy-1-fructosyl)valine, N-(1-deoxy-1-fructosyl) methionine, 1-[(5-amino-5-carboxypentyl)amino]-1-deoxyfructose, and dihydrolipoate) and their corresponding ROC curves. Data are represented as mean ± SD.

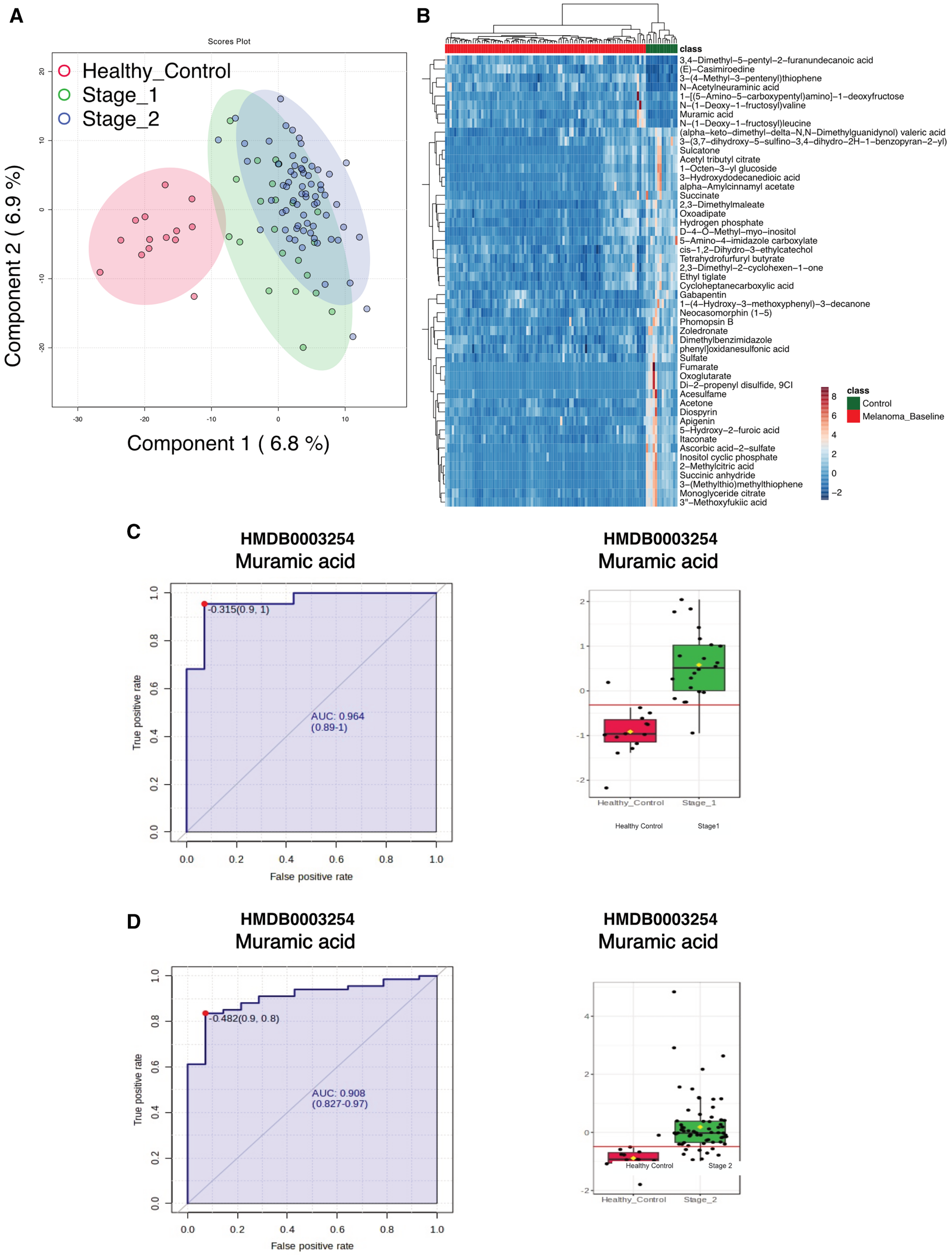

Figure 4. Serum metabolome data from a cohort consisting of patients with stage I and stage II melanoma further confirm the difference between healthy control individuals and patients with melanoma

(A) Partial least squares discriminant analysis (PLS-DA) showing a clear separation among 22 patients with stage I melanoma, 67 patients with stage II melanoma, and 14 healthy controls based on the 1,170 metabolites detected.

(B–D) (B) Heatmap showing the top 50 significant metabolite changes across melanoma and control individuals. Intensity levels of muramic acid in patients with (C) stage I and (D) stage II melanoma and the corresponding ROC curves. Data are shown as boxplots with median, interquartile range, and 1.5× IQR whiskers; individual observations are overlaid with jitter.

Our findings also highlighted significant changes in organic acid metabolism. Cancer cells, including melanoma cells, often exhibit altered energy metabolisms, particularly increased glycolysis and alterations in the tricarboxylic acid cycle. This metabolic reprogramming supports rapid cell growth and survival under various conditions, as corroborated by studies demonstrating modifications in amino acid and organic acid metabolism in melanoma.[27]

Specific pathway enrichments, such as ascorbate and alternate metabolism as well as D-glutamine and D-glutamate metabolism, in our analysis, may reflect the adaptation of melanoma cells to oxidative stress and an increased demand for amino acids for protein synthesis. Thus, our findings resonate with the research highlighting the importance of amino acid metabolism in cancers such as melanoma, which supports rapid growth and adaptation to the tumor microenvironment.[28]

Thus, identifying molecules related to such pathways as biomarkers for melanoma makes sense in a way that this might represent the presence of the malignant diseases/melanoma tumor in the individual. Based on these findings, we anticipate that the melanoma cells are frequently the origin of those molecules. This might also explain the altered levels of those molecules in patients with melanoma when compared to healthy individuals.

Muramic acid is an amino sugar acid and as such a bacterial wall/structural component and is part of the bacterial peptidoglycan. Thus, it originates from bacteria. However, indeed, it is less obvious why such bacterial wall component is present in such a high abundance in the serum of patients with melanoma. Of note, it has been demonstrated that simply the presence of the melanoma tumor might increase intestinal barrier defects,[29] thus promoting the occurrence of bacterial components in the patient's serum.[30]

On a functional level, muramic acid as a bacterial antigen might impact on, particularly, adaptive immune responses but potentially also affect cancer cell proliferation, cell death, or immunogenicity. However, of note, muramic acid is not specific to distinct bacterial species; thus, it might not indicate the presence of specific bacterial species contributing to melanoma development but rather indicate somehow a general mechanism of barrier leakage occurring in patients with melanoma. Nevertheless, one must admit that a simple intestinal barrier breakdown is not overly likely to be the cause of muramic acid in the serum of patients with melanoma, since muramic acid levels in patients with IBD or CRC, both being diseases that classically feature severe intestinal barrier disruption, are not altered when compared to that of healthy individuals. This suggests that there might indeed be a melanoma-specific mechanism and/or function of muramic acid. Gaining further functional understanding of the potential mechanistic role of muramic acid with respect to the pathogenesis of melanoma and its impact on the immune system will be part of our further research lines.

The consistent separation between melanoma and control groups in our PLS-DA mirrors the distinct metabolic profiles often observed in patients with cancer compared to healthy individuals.[13,31] This distinction in metabolic profiles is pivotal for the identification of potential biomarkers for early detection and monitoring of melanoma, as previous metabolomics studies suggested for various cancers.[32]

Bayci et al. had previously identified serum-based biomarkers for melanoma using targeted metabolomics.[21] However, in contrast to our data, by performing targeted metabolomics, they clearly limited their potential targets. Further, they analyzed a single cohort only consisting of 26 patients with melanoma. Thus, our data, generated by performing untargeted serum metabolomics, seem more relevant for the clinical setting, particularly since we additionally validated our findings from our much larger cohort of patients with melanoma in an independent validation cohort. Although the identification of stage-specific biomarkers is important, our current sample sizes within individual stages were not sufficient to allow for statistically robust stratified analyses. To address this limitation, we focused on identifying biomarkers that were consistently altered across cohorts and stages.

At the clinical level, our data provide evidence for a number of highly promising biomarkers for the diagnosis of melanoma. By featuring an AUC of at least 0.9544 in both the exploratory as well as the validation cohort, six of our identified biomarkers are able to accurately diagnose melanoma in a clinical setting. Particularly our lead biomarker, namely muramic acid, represents a very promising accuracy across all melanoma stages. Considering their AUC, they might be clearly superior to existing biomarkers such as S-100B or lactate dehydrogenase (LDH) in this regard. The discriminative ability of serum S-100B to identify disease relapse (pooled area under the ROC [AUROC] 78.64 [95% confidence interval (CI) 70.28; 87.01]) was significantly greater than the discriminative ability of serum LDH (AUROC 64.41 [95% CI 56.05; 7278]) (p = 0.013). Both are far lower than metabolite biomarkers described herein.[33]

Our serum biomarker could tremendously enhance the current diagnostic standards. First, our data suggest that the accuracy of our biomarker is outstandingly high, which will significantly enhance the accuracy of diagnosis and thus the detection rate of melanoma. Secondly, by performing an easy serum test, the screening using our biomarker can be easily performed across the population (at risk, but also not at risk) even by primary care physicians. Thus, the screening rates for melanoma would tremendously increase. Thirdly, the serum test would indicate the presence of melanoma. Thus, a positive serum test requires effort by the treating physician/dermatologist to identify the melanoma. Current screening strategies are dependent on visually detecting the melanoma, e.g., by dermoscopy. However, it can happen that, e.g., on the head or other regions of the body where visibility of the skin is restricted (e.g., by hairs, etc.), melanoma is not detected. Our biomarker would overcome this problem, since a positive test would require an extensive search for the melanoma until it has indeed been detected and found. Fourthly, several naevi/skin lesions can macroscopically look similar to melanomas, and frequently, histology needs to be taken to determine whether melanoma is present or not. Here, our biomarkers would tremendously help, since a positive serum screening test would support the decision-making process of the treating physician to indeed take out the suspicious lesion. Thus, overall, there are many factors explaining how our serum biomarker would tremendously enhance current diagnostic standards and improve patient care.

Overall, our data provide a comprehensive description of the entire serum metabolome composition in patients with melanoma. They demonstrate that patients with melanoma have a distinctly different metabolomics serum profile compared to healthy controls and that specific serum metabolites may serve as tumor markers for melanoma diagnosis, which could be of significant translational importance for patient care. We acknowledge that the identification of metabolites was based on accurate mass and database matching, which does not allow for definitive structural confirmation, especially in distinguishing isomers. Therefore, a limitation of this work is the absence of orthogonal validation using complementary analytical techniques.

Future studies should aim to validate the identity and diagnostic relevance of the key metabolites identified here using targeted approaches such as liquid chromatography-tandem mass spectrometry or NMR spectroscopy. These methods would enable unambiguous confirmation of metabolite structures and provide quantitative measurements, thereby enhancing the translational applicability of our findings. We have highlighted this as an important direction for follow-up investigations. While muramic acid levels clearly distinguished patients with melanoma from healthy controls in our main analysis, we observed no statistically significant differences between healthy individuals and patients with other diseases in these independent cohorts. Those findings suggest that muramic acid is not broadly altered across unrelated inflammatory or neoplastic conditions, thereby supporting its potential specificity for melanoma.

In conclusion, our study corroborates and extends the current understanding of melanoma metabolism, emphasizing the potential of metabolomics in unraveling the complex metabolic alterations associated with melanoma. These insights pave the way for the exploration of therapeutic targets and biomarkers, contributing to the evolving landscape of melanoma research and treatment.

Limitations of the study

While our study identifies six promising serum metabolite biomarkers for melanoma through an exploratory and validation cohort design, several limitations must be considered regarding the clinical utility and generalizability of our findings. First, the metabolomics profiling was performed using an Agilent QTOF 6550 mass spectrometer, which offers high-resolution data but cannot determine absolute metabolite concentrations. As a result, fold changes rather than absolute values were used for biomarker selection, which may limit direct clinical translation or threshold-based diagnostic development. Second, all serum samples were collected exclusively from the Zurich region. This geographic constraint may restrict the demographic and environmental diversity of the cohort. Expanding the study to include a more globally representative population is essential to ensure broader applicability and to develop robust, standardized biomarker panels suitable for global use. Finally, although we employed a two-phase discovery validation design, the control cohort was relatively small (n = 18). Further validation in larger, multi-center studies will be crucial to confirm the diagnostic performance, specificity, and clinical utility of the proposed biomarkers.

RESOURCE AVAILABILITY

Lead contact

Requests for further information, resources, and reagents should be directed to and will be fulfilled by the lead contact, Michael Scharl (michael.scharl@usz.ch).

Materials availability

This study did not generate new, unique reagents.

Data and code availability

- The datasets generated and/or analyzed during the current study are available from Zenodo: https://doi.org/10.5281/zenodo.15845162. Due to intellectual property considerations and patent-related restrictions, the data are not publicly available; however, they can be made accessible upon reasonable request and with permission from the corresponding author.
- The code was deposited in GitHub and is mentioned in the key resources table.
- Any additional information required to reanalyze the data reported in this work is available from the lead contact upon request.

ACKNOWLEDGMENTS

We thank Damina Balmer for providing editorial assistance.

This work was supported by the Lighthouse project grant of the Comprehensive Cancer Center Zurich (CCCZ) and University Medicine Zurich (UMZH), the Monique Dornonville de la Cour-Stiftung (M.S.), Stiftung Experimentelle Biomedizin (M.S.), Swiss National Science Foundation grant no. 320030_184753 (M.S.), Swiss National Science Foundation grant no. 320030E_190969 (M.S.), the Swiss Cancer Research Foundation grant no. KFS-5372-08-2021-R (M.S.), and the Fondazione San Salvatore (M.S.).

AUTHOR CONTRIBUTIONS

Conceptualization, M.S., Y.M., and M.P.L.; methodology, Y.M.; investigation, M.S., Y.M., M.P.L., B.H., P.T., M.B., J.M.M.-G., N.Z., G.R., and R.D.; visualization, Y.M.; funding acquisition, M.S.; project administration, M.S. and M.P.L.; supervision, M.S. and M.P.L.; writing – original draft, Y.M. and M.S.; and writing – review and editing, all authors.

DECLARATION OF INTERESTS

M.S. is a co-founder and shareholder of Recolony AG, Zurich, CH, and a shareholder of PharmaBiome AG, Zurich, CH. M.S. served as an advisor for AbbVie, Gilead, Fresenius, Topadur, Takeda, Roche, and Celltrion. M.S. received speaker's honoraria from Janssen, Falk Pharma, Vifor Pharma, PiLeJe, and Bromatech. M.S. received research grants from AbbVie, Takeda, Gilead, Gnubiotics, Roche, Axalbion, PharmaBiome, Topadur, Basilea, MBiomics, Storm Therapeutics, LimmaTech, Zealand Pharma, NodThera, Calypso Biotech, PiLeJe, Herbodee, and Vifor.

R.D. has intermittent, project-focused consulting and/or advisory relationships with Novartis, Merck Sharp & Dohme (MSD), Bristol Myers Squibb (BMS), Roche, Amgen, Takeda, Pierre Fabre, Sun Pharma, Sanofi, CatalYm, Second Genome, Regeneron, T3 Pharma, MaxiVAX SA, Pfizer, and Simcere outside the submitted work.

G.R. has consulted with AbbVie, Arena, Augurix, BMS, Boehringer, Calypso, Celgene, FALK, Ferring, Fisher, Genentech, Gilead, Janssen, Lilly, MSD, Novartis, Pfizer, Phadia, Roche, UCB, Takeda, Tillotts, Vifor, Vital Solutions, and Zeller. G.R. has received speaker's honoraria from AbbVie, AstraZeneca, BMS, Celgene, FALK, Janssen, MSD, Pfizer, Phadia, Takeda, Tillotts, UCB, Vifor, and Zeller. G.R. has received educational grants and research grants from AbbVie, Ardeypharm, Augurix, Calypso, FALK, Flamentera, MSD, Novartis, Pfizer, Roche, Takeda, Tillotts, UCB, and Zeller. G.R. is the co-founder and head of the scientific advisory board of PharmaBiome, a microbiota company.

STAR★METHODS

Detailed methods are provided in the online version of this paper and include the following:

- KEY RESOURCES TABLE
- EXPERIMENTAL MODEL AND STUDY PARTICIPANT DETAILS
  - Human subjects and ethical approval
- METHOD DETAILS
  - Serum collection and storage
  - Untargeted metabolomics analysis
  - Data preprocessing
- QUANTIFICATION AND STATISTICAL ANALYSIS
  - Biomarker evaluation via ROC curve

SUPPLEMENTAL INFORMATION

Supplemental information can be found online at https://doi.org/10.1016/j.xcrm.2025.102283.

Received: July 8, 2024
Revised: April 16, 2025
Accepted: July 11, 2025
Published: August 11, 2025

REFERENCES

1. Garbe, C., Keim, U., Gandini, S., Amaral, T., Katalinic, A., Hollezcek, B., Martus, P., Flatz, L., Leiter, U., and Whiteman, D. (2021). Epidemiology of cutaneous melanoma and keratinocyte cancer in white populations 1943-2036. Eur. J. Cancer *152*, 18–25. https://doi.org/10.1016/j.ejca.2021.04.029.
2. Paulson, K.G., Gupta, D., Kim, T.S., Veatch, J.R., Byrd, D.R., Bhatia, S., Wojcik, K., Chapuis, A.G., Thompson, J.A., Madeleine, M.M., and Gardner, J.M. (2020). Age-Specific Incidence of Melanoma in the United States. JAMA Dermatol. *156*, 57–64. https://doi.org/10.1001/jamadermatol.2019.3353.
3. Baade, P.D., Whiteman, D.C., Janda, M., Cust, A.E., Neale, R.E., Smithers, B.M., Green, A.C., Khosrotehrani, K., Mar, V., Soyer, H.P., and Aitken, J.F. (2020). Long-term deaths from melanoma according to tumor thickness at diagnosis. Int. J. Cancer *147*, 1391–1396. https://doi.org/10.1002/ijc.32930.
4. Arnold, M., de Vries, E., Whiteman, D.C., Jemal, A., Bray, F., Parkin, D.M., and Soerjomataram, I. (2018). Global burden of cutaneous melanoma attributable to ultraviolet radiation in 2012. Int. J. Cancer *143*, 1305–1314. https://doi.org/10.1002/ijc.31527.
5. Fidler, M.M., Gupta, S., Soerjomataram, I., Ferlay, J., Steliarova-Foucher, E., and Bray, F. (2017). Cancer incidence and mortality among young adults aged 20-39 years worldwide in 2012: a population-based study. Lancet Oncol. *18*, 1579–1589. https://doi.org/10.1016/S1470-2045(17)30677-0.
6. Arnold, M., Singh, D., Laversanne, M., Vignat, J., Vaccarella, S., Meheus, F., Cust, A.E., de Vries, E., Whiteman, D.C., and Bray, F. (2022). Global Burden of Cutaneous Melanoma in 2020 and Projections to 2040. JAMA Dermatol. *158*, 495–503. https://doi.org/10.1001/jamadermatol.2022.0160.
7. Deacon, D.C., Smith, E.A., and Judson-Torres, R.L. (2021). Molecular Biomarkers for Melanoma Screening, Diagnosis and Prognosis: Current State and Future Prospects. Front. Med. *8*, 642380. https://doi.org/10.3389/fmed.2021.642380.
8. Weide, B., Elsässer, M., Büttner, P., Pflugfelder, A., Leiter, U., Eigentler, T. K., Bauer, J., Witte, M., Meier, F., and Garbe, C. (2012). Serum markers lactate dehydrogenase and S100B predict independently disease outcome in melanoma patients with distant metastasis. Br. J. Cancer *107*, 422–428. https://doi.org/10.1038/bjc.2012.306.
9. Gershenwald, J.E., Scolyer, R.A., Hess, K.R., Sondak, V.K., Long, G.V., Ross, M.I., Lazar, A.J., Faries, M.B., Kirkwood, J.M., McArthur, G.A., et al. (2017). Melanoma staging: Evidence-based changes in the American Joint Committee on Cancer eighth edition cancer staging manual. CA Cancer J. Clin. *67*, 472–492. https://doi.org/10.3322/caac.21409.
10. Gopalakrishnan, V., Spencer, C.N., Nezi, L., Reuben, A., Andrews, M.C., Karpinets, T.V., Prieto, P.A., Vicente, D., Hoffman, K., Wei, S.C., et al. (2018). Gut microbiome modulates response to anti-PD-1 immunotherapy in melanoma patients. Science *359*, 97–103. https://doi.org/10.1126/science.aan4236.
11. Routy, B., Le Chatelier, E., Derosa, L., Duong, C.P.M., Alou, M.T., Daillère, R., Fluckiger, A., Messaoudene, M., Rauber, C., Roberti, M.P., et al. (2018). Gut microbiome influences efficacy of PD-1-based immunotherapy against epithelial tumors. Science *359*, 91–97. https://doi.org/10.1126/science.aan3706.
12. Vetizou, M., Pitt, J.M., Daillere, R., Lepage, P., Waldschmitt, N., Flament, C., Rusakiewicz, S., Routy, B., Roberti, M.P., Duong, C.P., et al. (2015). Anticancer immunotherapy by CTLA-4 blockade relies on the gut microbiota. Science *350*, 1079–1084. https://doi.org/10.1126/science.aad1329.
13. Weber, D.D., Thapa, M., Aminzadeh-Gohari, S., Redtenbacher, A.S., Catalano, L., Feichtinger, R.G., Koelblinger, P., Dallmann, G., Emberger, M., Kofler, B., and Lang, R. (2021). Targeted Metabolomics Identifies Plasma Biomarkers in Mice with Metabolically Heterogeneous Melanoma Xenografts. Cancers (Basel) *13*, 434. https://doi.org/10.3390/cancers13030434.
14. Taylor, N.J., Gaynanova, I., Eschrich, S.A., Welsh, E.A., Garrett, T.J., Beecher, C., Sharma, R., Koomen, J.M., Smalley, K.S.M., Messina, J.L., and Kanetsky, P.A. (2020). Metabolomics of primary cutaneous melanoma and matched adjacent extratumoral microenvironment. PLoS One *15*, e0240849. https://doi.org/10.1371/journal.pone.0240849.
15. Du, J., Su, Y., Qian, C., Yuan, D., Miao, K., Lee, D., Ng, A.H.C., Wijker, R.S., Ribas, A., Levine, R.D., et al. (2020). Raman-guided subcellular pharmaco-metabolomics for metastatic melanoma cells. Nat. Commun. *11*, 4830. https://doi.org/10.1038/s41467-020-18376-x.
16. Kim, H.Y., Lee, H., Kim, S.H., Jin, H., Bae, J., and Choi, H.K. (2017). Discovery of potential biomarkers in human melanoma cells with different metastatic potential by metabolic and lipidomic profiling. Sci. Rep. *7*, 8864. https://doi.org/10.1038/s41598-017-08433-9.
17. Shi, X., Tasdogan, A., Huang, F., Hu, Z., Morrison, S.J., and DeBerardinis, R.J. (2017). The abundance of metabolites related to protein methylation correlates with the metastatic capacity of human melanoma xenografts. Sci. Adv. *3*, eaao5268. https://doi.org/10.1126/sciadv.aao5268.
18. Frankel, A.E., Coughlin, L.A., Kim, J., Froehlich, T.W., Xie, Y., Frenkel, E.P., and Koh, A.Y. (2017). Metagenomic Shotgun Sequencing and Unbiased Metabolomic Profiling Identify Specific Human Gut Microbiota and Metabolites Associated with Immune Checkpoint Therapy Efficacy in Melanoma Patients. Neoplasia *19*, 848–855. https://doi.org/10.1016/j.neo.2017.08.004.
19. Bui, A., Choi, Y., Frankel, A.E., and Koh, A.Y. (2021). Unbiased Microbiome and Metabolomic Profiling of Fecal Samples from Patients with Melanoma. Methods Mol. Biol. *2265*, 461–474. https://doi.org/10.1007/978-1-0716-1205-7_33.
20. Vandoni, G., D'Amico, F., Fabbrini, M., Mariani, L., Sieri, S., Casirati, A., Di Guardo, L., Del Vecchio, M., Anichini, A., Mortarini, R., et al. (2023). Gut Microbiota, Metabolome, and Body Composition Signatures of Response to Therapy in Patients with Advanced Melanoma. Int. J. Mol. Sci. *24*, 11611. https://doi.org/10.3390/ijms241411611.
21. Bayci, A.W.L., Baker, D.A., Somerset, A.E., Turkoglu, O., Hothem, Z., Callahan, R.E., Mandal, R., Han, B., Bjorndahl, T., Wishart, D., et al. (2018). Metabolomic identification of diagnostic serum-based biomarkers for advanced stage melanoma. Metabolomics *14*, 105. https://doi.org/10.1007/s11306-018-1398-9.

CellPress
OPEN ACCESS

22. Pellerin, L., Carrie, L., Dufau, C., Nieto, L., Segui, B., Levade, T., Riond, J., and Andrieu-Abadie, N. (2020). Lipid metabolic Reprogramming: Role in Melanoma Progression and Therapeutic Perspectives. Cancers (Basel) *12*, 3147. https://doi.org/10.3390/cancers12113147.

23. Fahrmann, J.F., Vykoukal, J.V., and Ostrin, E.J. (2020). Amino Acid Oncometabolism and Immunomodulation of the Tumor Microenvironment in Lung Cancer. Front. Oncol. *10*, 276. https://doi.org/10.3389/fonc.2020.00276.

24. Rohrig, F., and Schulze, A. (2016). The multifaceted roles of fatty acid synthesis in cancer. Nat. Rev. Cancer *16*, 732–749. https://doi.org/10.1038/nrc.2016.89.

25. Henderson, F., Johnston, H.R., Badrock, A.P., Jones, E.A., Forster, D., Nagaraju, R.T., Evangelou, C., Kamarashev, J., Green, M., Fairclough, M., et al. (2019). Enhanced Fatty Acid Scavenging and Glycerophospholipid Metabolism Accompany Melanocyte Neoplasia Progression in Zebrafish. Cancer Res. *79*, 2136–2151. https://doi.org/10.1158/0008-5472.CAN-18-2409.

26. Srivastava, A., Srivastava, P., Mathur, S., Abbas, S., Rai, N., Tiwari, S., Tiwari, M., and Sharma, L. (2022). Lipid Metabolism and Mitochondria: Cross Talk in Cancer. Curr. Drug Targets *23*, 606–627. https://doi.org/10.2174/1389450122666210824144907.

27. DeBerardinis, R.J., and Chandel, N.S. (2016). Fundamentals of cancer metabolism. Sci. Adv. *2*, e1600200. https://doi.org/10.1126/sciadv.1600200.

28. Kosmopoulou, M., Giannopoulou, A.F., Iliou, A., Benaki, D., Panagiotakis, A., Velentzas, A.D., Konstantakou, E.G., Papassideri, I.S., Mikros, E., Stravopodis, D.J., and Gikas, E. (2020). Human Melanoma-Cell Metabolic Profiling: Identification of Novel Biomarkers Indicating Metastasis. Int. J. Mol. Sci. *21*, 2436. https://doi.org/10.3390/ijms21072436.

29. Yonekura, S., Terrisse, S., Alves Costa Silva, C., Lafarge, A., Iebba, V., Ferrere, G., Goubet, A.G., Fahrner, J.E., Lahmar, I., Ueda, K., et al. (2022). Cancer Induces a Stress Ileopathy Depending on beta-Adrenergic Receptors and Promoting Dysbiosis that Contributes to Carcinogenesis. Cancer Discov. *12*, 1128–1151. https://doi.org/10.1158/2159-8290.CD-21-0999.

30. Routy, B., Jackson, T., Mählmann, L., Baumgartner, C.K., Blaser, M., Byrd, A., Corvaia, N., Couts, K., Davar, D., Derosa, L., et al. (2024). Melanoma and microbiota: Current understanding and future directions. Cancer Cell *42*, 16–34. https://doi.org/10.1016/j.ccell.2023.12.003.

31. Gil, J., Kim, Y., Szeitz, B., Doma, V., Çakır, U., de Almeida, N.P., Hagemeijer, Y.P., Guryev, V., Johansson, J.G., Sharma, Y., et al. (2021). Proteogenomics Reveals how Metastatic Melanoma Modulates the Immune System to Allow Immune Evasion. Preprint at bioRxiv. https://doi.org/10.1101/2021.04.10.439245.

32. Wishart, D.S. (2016). Emerging applications of metabolomics in drug discovery and precision medicine. Nat. Rev. Drug Discov. *15*, 473–484. https://doi.org/10.1038/nrd.2016.32.

33. Janka, E.A., Várvölgyi, T., Sipos, Z., Soós, A., Hegyi, P., Kiss, S., Dembrovszky, F., Csupor, D., Kéringer, P., Pécsi, D., et al. (2021). Predictive Performance of Serum S100B Versus LDH in Melanoma Patients: A Systematic Review and Meta-Analysis. Front. Oncol. *11*, 772165. https://doi.org/10.3389/fonc.2021.772165.

34. Fuhrer, T., Heer, D., Begemann, B., and Zamboni, N. (2011). High-throughput, accurate mass metabolome profiling of cellular extracts by flow injection-time-of-flight mass spectrometry. Anal. Chem. *83*, 7074–7080. https://doi.org/10.1021/ac201267k.

35. Pang, Z., Chong, J., Li, S., and Xia, J. (2020). MetaboAnalystR 3.0: Toward an Optimized Workflow for Global Metabolomics. Metabolites *10*, 186. https://doi.org/10.3390/metabo10050186.

STAR★METHODS

KEY RESOURCES TABLE

REAGENT or RESOURCE	SOURCE	IDENTIFIER
Deposited data		
Metabolomics Data	This Paper	Zenodo: https://zenodo.org/records/15845162
Software and algorithms		
R v 4.0.0	The R Project for Statistical Computing	https://www.r-project.org
Other		
Agilent QTOF 6550 mass spectrometer	Agilent Technologies	https://www.agilent.com/en/products/liquid-chromatography
The Code used for the analysis	This paper	https://github.com/ymorsy/The-serum-metabolome-discriminates-patients-with-melanoma

EXPERIMENTAL MODEL AND STUDY PARTICIPANT DETAILS

Human subjects and ethical approval

Ethical approval for collecting serum samples as well as clinical characteristics from patients with melanoma and healthy control individuals after providing written informed consent was received by the Cantonal Ethics Committee of the Canton Zürich (EK647-PB_2018-00194, EK-1755-PB_2019-00169 and EK-2019-02277). Further use of the biosamples and clinical data for the specific project was granted by the Cantonal Ethics Committee of the Canton Zürich via approval KEK-ZH 2019-01337. All patients were recruited at the University Hospital Zurich (USZ) and diagnosed either with melanoma diagnosed with Union for International Cancer Control (UICC) stages I-IV stage I ($n = 22$), stage II ($n = 67$), stage III ($n = 65$), stage IV ($n = 59$), Inflammatory Bowel Disease (IBD, $n = 12$), Colorectal Cancer (CRC, $n = 28$), Psoriasis ($n = 12$) or Squamous Cell Skin Cancer (SSC, $n = 9$). The sample size used in the described activity was determined based on the availability of clinical samples. Patients at a minimum of 18 years of age were selected with a balanced distribution of age and gender. After obtaining written informed consent from the patients, respective specimens and clinical data were collected. Additionally, overall, 32 control individuals were recruited from the outpatient clinic of the Department of Gastroenterology and Hepatology, University Hospital Zurich.

METHOD DETAILS

Serum collection and storage

Blood was collected in non-coated vacutainers, and then centrifuged at 2000 g for 10 min at RT. Subsequently obtained serum was snap frozen and stored at -80°C until further processing.

Untargeted metabolomics analysis

Metabolomics analysis was carried out by flow-injection mass spectrometry on Agilent QTOF 6550 as described previously by Fuhrer et al.,[34] The electrospray ionization was performed in both positive and negative modes. In positive ionization mode, the mobile phase was methanol/water (60:40, v/v) with 0.1% formic acid (pH 3). In negative mode, the mobile phase consisted of isopropanol/water (60:40, v/v) buffered with 5 mM ammonium carbonate (pH 9). The flow rate was maintained at 150 μL/min, with sample injection volume of 1.6 μL in full-loop mode. The electrospray ionization was performed in both positive and negative modes. In positive ionization mode, the mobile phase was methanol/water (60:40, v/v) with 0.1% formic acid (pH 3). In negative mode, the mobile phase consisted of isopropanol/water (60:40, v/v) buffered with 5 mM ammonium carbonate (pH 9). The flow rate was maintained at 150 μL/min, with sample injection volume of 1.6 μL in full-loop mode. We used MetaboAnalystR package version 4.0.0 for the statistical analysis.[35]

Data preprocessing

Regarding quality control, our experimental workflow followed well-established protocols, including standardized metabolite extraction procedures, rigorous QC checks, and systematic data acquisition. Quality was objectively monitored using coefficient of variation (CV) and relative standard deviation (RSD) values for total ion current (TIC) and detected features. The consistently low CVs within each group confirm the robustness and reproducibility of our measurements, supporting confidence in the downstream statistical analyses.

Missing and zero values were handled using the default imputation method provided in the MetaboAnalyst R package. This method replaces such values with a small constant equal to half of the minimum positive value observed in the dataset, based on the assumption that most missing values are due to low-abundance metabolites falling below the detection limit. Following imputation, we applied interquartile range (IQR)-based filtering to remove features with low variability across samples. The normalized intensities by the median for each sample were mean-centered and divided by the standard deviation of each variable of all annotated features.

QUANTIFICATION AND STATISTICAL ANALYSIS

Significance analysis was done by heteroscedastic (two tail, unequal variance) *t*-test. Additionally, *p*-values were adjusted for the false discovery rate. The actual statistical values are provided in the figures and their corresponding legends. Partial Least Squares - Discriminant Analysis was used to visualize sample variance. Heatmap visually presented the hierarchical clustering using the Euclidean method for distance calculation and Ward's linkage for clustering. Metabolite Set Enrichment Analysis (MSEA) and pathway enrichment analysis were conducted using the MetaboAnalystR package. Enrichment was performed using the "main" metabolite set library of MetaboAnalyst, which contains curated main chemical structure metabolite sets. Enrichment analysis using a generalized linear model (GLM) was conducted to quantify the significance of enrichment scores per metabolite group. Significant metabolites were mapped to KEGG metabolic pathways and evaluated using the hypergeometric test in combination with pathway topology analysis using relative betweenness centrality. Metabolites were stratified first by upregulation and downregulation using fold-change and statistical significance to provide more meaningful biological context and interpretability. Enrichment analysis was conducted in the distinct categories of up and down to define the directionality of the enrichment.

Biomarker evaluation via ROC curve

Classical Receiver Operating Characteristic (ROC) curve analysis was conducted using the pROC package in R to measure the efficacy of the chosen metabolites as a biomarker in distinguishing melanoma patients and healthy donors. We want to highlight that the two cohorts were not combined to discover the biomarker. Still, rather both cohorts were analyzed individually to look at metabolites that had high discriminatory power (AUC >0.95) and might serve as potential biomarkers. We next looked at the overlap of top-performing metabolites in both cohorts that were highly significant in both the exploratory and validation cohort.

Cell Reports Medicine, Volume *6*

Supplemental information

The serum metabolome serves as a diagnostic biomarker and discriminates patients with melanoma from healthy individuals

Yasser Morsy, Barbara Hubeli, Patrick Turko, Marjam Barysch, Julia M. Martínez-Gómez, Nicola Zamboni, Gerhard Rogler, Reinhard Dummer, Mitchell P. Levesque, and Michael Scharl

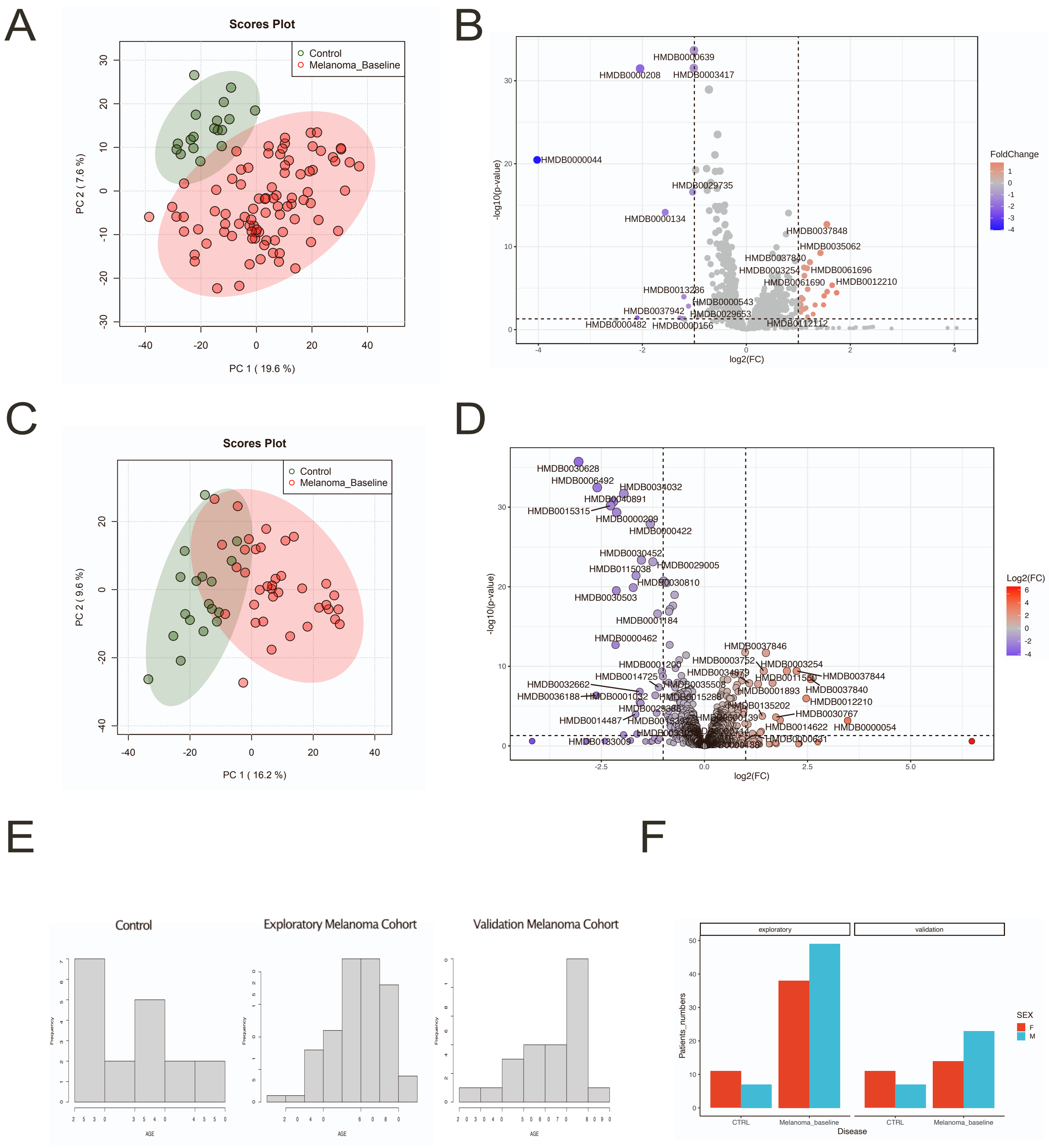

Supplementary Figure 1. Exploratory and validation cohort dimension reduction and volcano plot. Related to Figure 1 and 2 (A) Principle component analysis (PCA) showing a clear separation of the exploratory cohort between 87 melanoma patients and 18 healthy controls based on the metabolites detected. Every dot represents one patient. (B) Volcano plot showing the log2 fold change on the x-axis and -log10 p-value on the y-axis. (C) PCA showing a clear separation of the validation cohort between 37 melanoma patients and 18 healthy controls based on the metabolites detected. Every dot represents one patient. (D) Volcano plot showing the log2 fold change on the x-axis and -log10 p-value on the y-axis. (E) The number of patients categorized by age across the controls and melanoma cohorts. (F) The number of patients categorized by sex across the controls and melanoma cohorts.

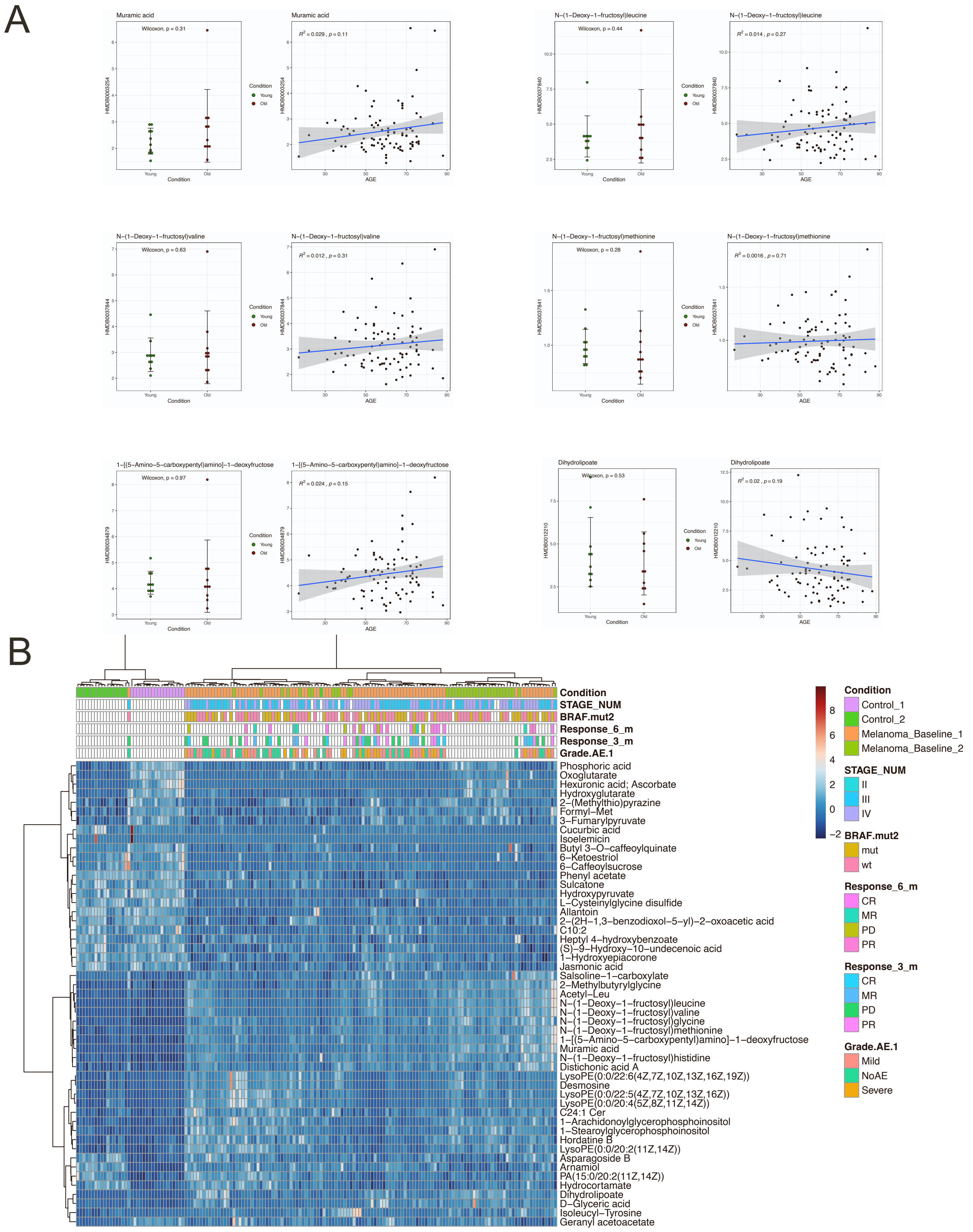

Supplementary Figure 2. Clinical data correlation with biomarkers of patients with stage III or IV melanoma. Related to Figure 3 (A) Ten patients were selected to represent the young patients, and another 10 represented the old patients to check statistics using the Wilcoxon test. For each metabolite, a regression analysis was carried out between the metabolite intensities and patients' ages. Muramic acid, leucine, valine, methionine, deoxyfructose, Dihydrolipoate.Data are represented as mean +/- SD (B) Annotated heatmap showing the absence of clustering based on any clinical data (tumor stage, BRAF mutation, Adverse events, and response after three or six months).

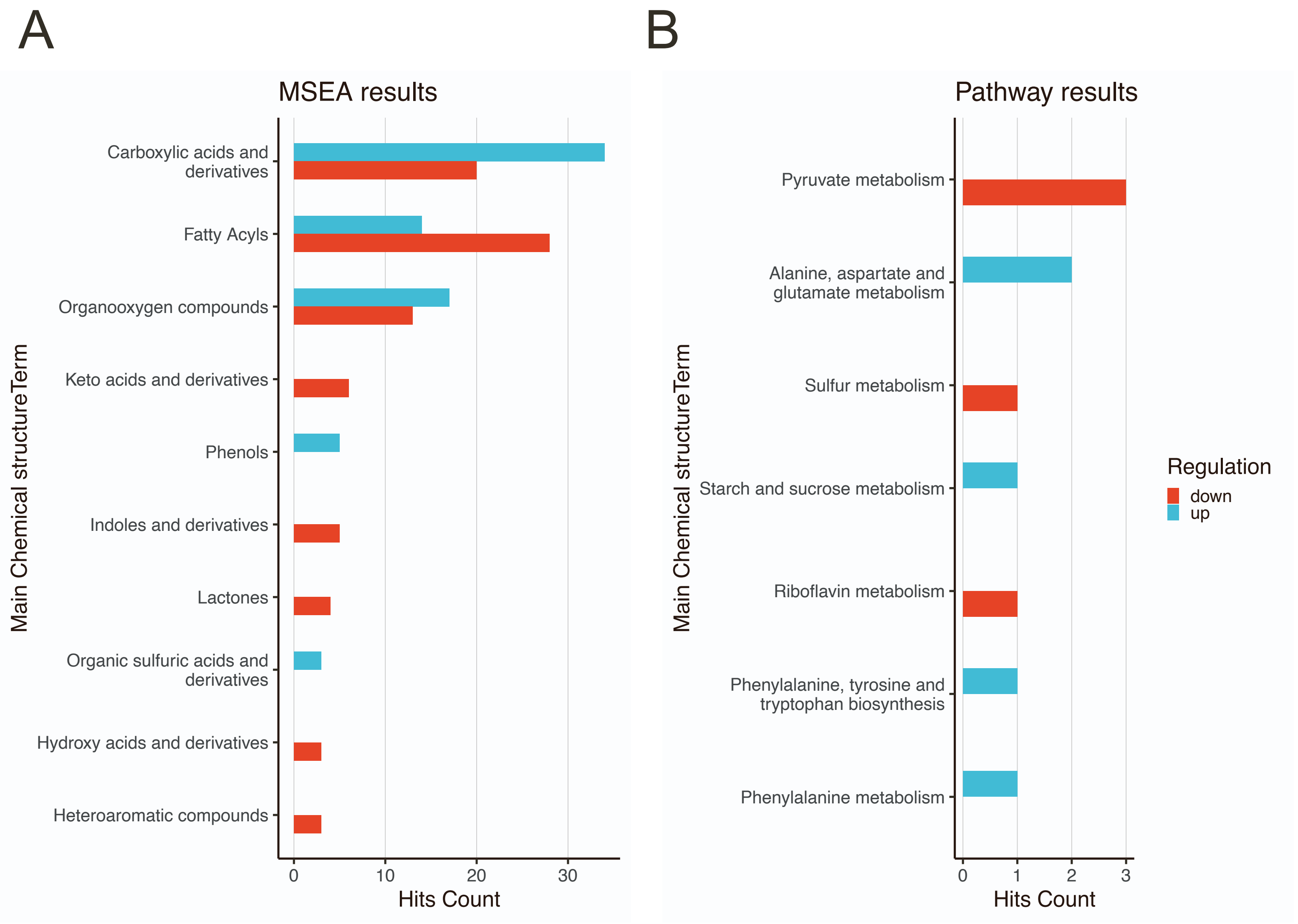

Supplementary Figure 3. Metabolite profiling demonstrates differences in enriched metabolite sets and affects pathways within the serum metabolome between healthy control individuals and patients with early stage melanoma. Related to Figure 4 (A) Bar graph showing top 25 affected metabolite sets after performing metabolite set enrichment analysis (MSEA). (B) Pathway enrichment analysis showing the most affected pathways ranked by the impact of the metabolites in these pathways.

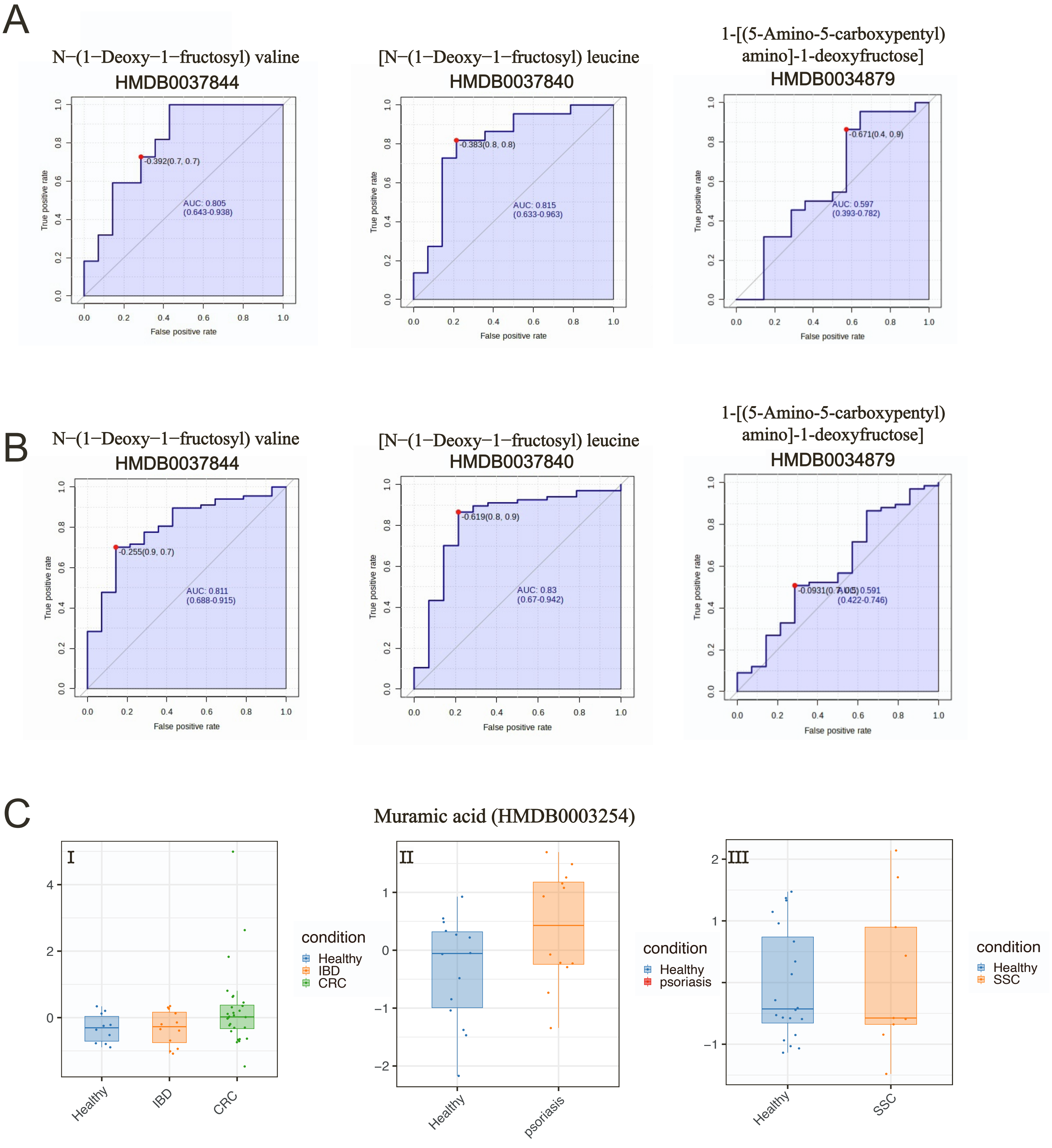

Supplementary Figure 4. Potential serum biomarkers for the diagnosis of patients with melanoma. Related to Figure 4
ROC curves of three top selected metabolites N−(1−Deoxy−1−fructosyl) valine(HMDB0037844), [N−(1−Deoxy−1−fructosyl) leucine (HMDB0037840), 1-[(5-Amino-5-carboxypentyl) amino]-1-deoxyfructose] (HMDB0034879) (A) stage I and (B) stage II patients with melanoma.(C) Centered and scaled levels of Muramic acid (HMDB0003254) across three independent validation cohorts. Comparisons among (I) healthy individuals (n=10) and patients with either Inflammatory Bowel Disease (IBD, n=12) or Colorectal Cancer (CRC, n=28), (II) Healthy individuals (n=14) and patients with Psoriasis (n=12), (III) Healthy individuals (n=20) and patients with Squamous Cell Skin Cancer (SSC, n=9).Data are shown as boxplots with median, interquartile range, and 1.5× IQR whiskers; individual observations are overlaid with jitter.

Supplementary table 1: Patient characteristics of the exploratory and validation cohort Related to Figure 1 and 2

ID_Metabolomics	Disease	SEX	AGE	STAGE_NUM	Cohort
ID1	Melanoma_baseline	F	60	III	exploratory
ID2	Melanoma_baseline	M	57	IV	exploratory
ID3	Melanoma_baseline	F	72	III	exploratory
ID4	Melanoma_baseline	M	54	III	exploratory
ID5	Melanoma_baseline	M	68	IV	exploratory
ID6	Melanoma_baseline	M	72	IV	exploratory
ID7	Melanoma_baseline	M	60	IV	exploratory
ID8	Melanoma_baseline	M	57	III	exploratory
ID9	Melanoma_baseline	F	22	IV	exploratory
ID10	Melanoma_baseline	M	59	III	exploratory
ID11	Melanoma_baseline	M	54	III	exploratory
ID12	Melanoma_baseline	F	65	IV	exploratory
ID13	Melanoma_baseline	M	66	IV	exploratory
ID15	Melanoma_baseline	M	60	IV	exploratory
ID16	Melanoma_baseline	F	58	III	exploratory
ID17	Melanoma_baseline	F	72	III	exploratory
ID18	Melanoma_baseline	F	52	III	exploratory
ID19	Melanoma_baseline	M	38	III	exploratory
ID20	Melanoma_baseline	F	64	IV	exploratory
ID21	Melanoma_baseline	F	40	IV	exploratory
ID22	Melanoma_baseline	M	45	IV	exploratory
ID23	Melanoma_baseline	F	58	III	exploratory
ID24	Melanoma_baseline	M	76	III	exploratory
ID25	Melanoma_baseline	F	38	III	exploratory
ID26	Melanoma_baseline	M	31	II	exploratory
ID27	Melanoma_baseline	F	83	IV	exploratory
ID28	Melanoma_baseline	M	60	III	exploratory
ID29	Melanoma_baseline	F	62	III	exploratory
ID30	Melanoma_baseline	M	68	III	exploratory
ID31	Melanoma_baseline	M	68	IV	exploratory
ID32	Melanoma_baseline	F	70	IV	exploratory
ID33	Melanoma_baseline	F	34	III	exploratory
ID34	Melanoma_baseline	F	35	III	exploratory
ID35	Melanoma_baseline	F	67	III	exploratory
ID36	Melanoma_baseline	M	41	III	exploratory
ID37	Melanoma_baseline	M	58	III	exploratory
ID38	Melanoma_baseline	F	73	III	exploratory
ID39	Melanoma_baseline	F	77	III	exploratory
ID40	Melanoma_baseline	M	67	IV	exploratory
ID41	Melanoma_baseline	M	68	III	exploratory
ID42	Melanoma_baseline	M	48	IV	exploratory
ID43	Melanoma_baseline	M	50	IV	exploratory
ID44	Melanoma_baseline	M	61	III	exploratory
ID45	Melanoma_baseline	M	64	III	exploratory
ID47	Melanoma_baseline	F	53	IV	exploratory
ID49	Melanoma_baseline	F	75	IV	exploratory
ID50	Melanoma_baseline	F	64	III	exploratory
ID51	Melanoma_baseline	F	72	III	exploratory
ID52	Melanoma_baseline	M	49	III	exploratory
ID53	Melanoma_baseline	M	56	IV	exploratory
ID54	Melanoma_baseline	M	56	III	exploratory
ID55	Melanoma_baseline	F	76	III	exploratory
ID56	Melanoma_baseline	M	54	III	exploratory
ID57	Melanoma_baseline	M	70	IV	exploratory
ID58	Melanoma_baseline	M	72	III	exploratory
ID60	Melanoma_baseline	F	49	IV	exploratory
ID61	Melanoma_baseline	M	73	III	exploratory
ID63	Melanoma_baseline	M	84	IV	exploratory
ID64	Melanoma_baseline	M	44	IV	exploratory
ID65	Melanoma_baseline	F	50	III	exploratory
ID66	Melanoma_baseline	M	61	IV	exploratory
ID67	Melanoma_baseline	M	70	III	exploratory
ID68	Melanoma_baseline	F	42	III	exploratory
ID69	Melanoma_baseline	M	53	IV	exploratory
ID70	Melanoma_baseline	F	73	III	exploratory
ID71	Melanoma_baseline	M	72	IV	exploratory

ID72	Melanoma_baseline	F	46	IV	exploratory
ID73	Melanoma_baseline	F	53	III	exploratory
ID74	Melanoma_baseline	F	73	IV	exploratory
ID75	Melanoma_baseline	M	75	IV	exploratory
ID76	Melanoma_baseline	M	64	IV	exploratory
ID77	Melanoma_baseline	M	39	IV	exploratory
ID78	Melanoma_baseline	F	88	IV	exploratory
ID79	Melanoma_baseline	M	55	IV	exploratory
ID80	Melanoma_baseline	F	68	III	exploratory
ID81	Melanoma_baseline	F	53	III	exploratory
ID82	Melanoma_baseline	F	17	IV	exploratory
ID83	Melanoma_baseline	F	68	III	exploratory
ID85	Melanoma_baseline	M	75	III	exploratory
ID86	Melanoma_baseline	M	61	III	exploratory
ID87	Melanoma_baseline	M	54	IV	exploratory
ID88	Melanoma_baseline	M	83	IV	exploratory
ID89	Melanoma_baseline	M	75	III	exploratory
ID90	Melanoma_baseline	F	66	III	exploratory
ID91	Melanoma_baseline	M	35	III	exploratory
ID92	Melanoma_baseline	F	48	IV	exploratory
ID93	Melanoma_baseline	M	73	III	exploratory
ID119	CTRL	F	29	NA	exploratory
ID120	CTRL	F	30	NA	exploratory
ID121	CTRL	F	37	NA	exploratory
ID122	CTRL	M	34	NA	exploratory
ID123	CTRL	M	44	NA	exploratory
ID124	CTRL	M	40	NA	exploratory
ID125	CTRL	M	36	NA	exploratory
ID126	CTRL	F	40	NA	exploratory
ID127	CTRL	F	49	NA	exploratory
ID128	CTRL	F	27	NA	exploratory
ID129	CTRL	M	26	NA	exploratory
ID132	CTRL	F	43	NA	exploratory
ID133	CTRL	F	26	NA	exploratory
ID134	CTRL	M	30	NA	exploratory
ID135	CTRL	F	37	NA	exploratory
ID136	CTRL	M	29	NA	exploratory
ID137	CTRL	F	48	NA	exploratory
ID138	CTRL	F	32	NA	exploratory
ID_199	Melanoma_baseline	M	76	IV	validation
ID_200	Melanoma_baseline	M	78	IV	validation
ID_201	Melanoma_baseline	F	41	III	validation
ID_202	Melanoma_baseline	M	71	IV	validation
ID_203	Melanoma_baseline	F	52	missing	validation
ID_204	Melanoma_baseline	F	83	IV	validation
ID_205	Melanoma_baseline	M	47	III	validation
ID_206	Melanoma_baseline	M	62	IV	validation
ID_207	Melanoma_baseline	M	70	III	validation
ID_208	Melanoma_baseline	F	38	IV	validation
ID_209	Melanoma_baseline	M	52	III	validation
ID_210	Melanoma_baseline	F	54	IV	validation
ID_211	Melanoma_baseline	M	76	III	validation
ID_212	Melanoma_baseline	F	77	III	validation
ID_213	Melanoma_baseline	M	56	III	validation
ID_214	Melanoma_baseline	M	76	IV	validation
ID_215	Melanoma_baseline	M	59	IV	validation
ID_216	Melanoma_baseline	F	59	III	validation
ID_217	Melanoma_baseline	M	79	IV	validation
ID_218	Melanoma_baseline	M	46	IV	validation
ID_219	Melanoma_baseline	M	64	IV	validation
ID_220	Melanoma_baseline	F	77	III	validation
ID_221	Melanoma_baseline	F	80	IV	validation
ID_222	Melanoma_baseline	M	76	III	validation
ID_224	Melanoma_baseline	F	76	III	validation
ID_225	Melanoma_baseline	F	54	III	validation
ID_226	Melanoma_baseline	F	61	IV	validation
ID_227	Melanoma_baseline	M	67	IV	validation
ID_228	Melanoma_baseline	M	65	IV	validation
ID_229	Melanoma_baseline	M	70	IV	validation

ID_231	Melanoma_baseline	M	30	IV	validation
ID_232	Melanoma_baseline	M	78	IV	validation
ID_233	Melanoma_baseline	M	70	IV	validation
ID_234	Melanoma_baseline	F	64	III	validation
ID_235	Melanoma_baseline	M	91	III	validation
ID_236	Melanoma_baseline	M	83	III	validation
ID_237	Melanoma_baseline	F	60	IV	validation
ID119	CTRL	F	29	NA	validation
ID120	CTRL	F	30	NA	validation
ID121	CTRL	F	37	NA	validation
ID122	CTRL	M	34	NA	validation
ID123	CTRL	M	44	NA	validation
ID124	CTRL	M	40	NA	validation
ID125	CTRL	M	36	NA	validation
ID126	CTRL	F	40	NA	validation
ID127	CTRL	F	49	NA	validation
ID128	CTRL	F	27	NA	validation
ID129	CTRL	M	26	NA	validation
ID132	CTRL	F	43	NA	validation
ID133	CTRL	F	26	NA	validation
ID134	CTRL	M	30	NA	validation
ID135	CTRL	F	37	NA	validation
ID136	CTRL	M	29	NA	validation
ID137	CTRL	F	48	NA	validation
ID138	CTRL	F	32	NA	validation

Supplementary table 3: Altered metabolites with fold change above 2 or below 0.5 Related to Figure 1					
ID	Name	FC	log2(FC)	p.ajusted	LOG10(p)
HMDB0011487	LysoPE(0:0/20:4(5Z,8Z,11Z,14Z))	3.3327	1.7367	3.54E-05	4.4507
HMDB0012210	Dihydrolipoate	3.1393	1.6505	4.43E-06	5.3534
HMDB0011494	LysoPE(0:0/22:5(4Z,7Z,10Z,13Z,16Z))	2.9357	1.5537	2.64E-05	4.5785
HMDB0037848	N-(1-Deoxy-1-fructosyl)glycine	2.9247	1.5483	1.99E-13	12.702
HMDB0011485	LysoPE(0:0/20:3(5Z,8Z,11Z))	2.8213	1.4964	8.51E-05	4.0702
HMDB0011496	LysoPE(0:0/22:6(4Z,7Z,10Z,13Z,16Z,19Z))	2.7988	1.4848	0.001028	2.988
HMDB0035062	Arnamiol	2.6853	1.4251	5.65E-10	9.2479
HMDB0000572	Desmosine	2.5008	1.3224	0.0010576	2.9757
HMDB0029369	Neosaxitoxin	2.4364	1.2847	0.01334	1.8748
HMDB0037840	N-(1-Deoxy-1-fructosyl)leucine	2.3352	1.2235	7.27E-09	8.1383
HMDB0000139	D-Glyceric acid	2.2644	1.1791	1.35E-05	4.8682
HMDB0112112	FAHFA(18:0/9-O-18:0)	2.2584	1.1753	0.028059	1.5519
HMDB0061696	1-Stearoylglycerophosphoinositol	2.2451	1.1668	3.90E-08	7.4091
HMDB0011479	LysoPE(0:0/18:3(9Z,12Z,15Z))	2.2128	1.1459	0.0024223	2.6158
HMDB0061690	1-Arachidonoylglycerophosphoinositol	2.1683	1.1166	3.15E-07	6.5014
HMDB0003254	Muramic acid	2.1589	1.1103	3.20E-08	7.4943
HMDB0009215	PE(18:4(6Z,9Z,12Z,15Z)/P-18:1(11Z))	2.1576	1.1095	0.0034943	2.4566
HMDB0011473	LysoPE(0:0/16:0)	2.127	1.0888	0.0002018	3.6951
HMDB0002925	8,11,14-Eicosatrienoic acid	2.0816	1.0577	0.0001331	3.8759
HMDB0001976	C22:5	2.0732	1.0519	0.0020444	2.6894
HMDB0008984	PE(16:1(9Z)/P-18:1(11Z))	2.0712	1.0504	0.0009435	3.0253
HMDB0029854	1,2-Epoxy-1,2,7,7'',8,8'',11'',12''-octahydro-psi,psi-carotene	2.0687	1.0487	0.008059	2.0937
HMDB0002226	C22:4	2.0511	1.0364	0.0006779	3.1688
HMDB0000044	Hexuronic acid; Ascorbate	0.061426	-4.025	3.44E-21	20.464
HMDB0000482	Octanoic acid	0.23347	-2.0987	0.036361	1.4394
HMDB0000208	Oxoglutarate	0.24249	-2.044	3.35E-32	31.475
HMDB0000134	Fumarate	0.33903	-1.5605	7.08E-15	14.15
HMDB0037942	(1xi,3xi)-1,2,3,4-Tetrahydro-1-methyl-beta-carboline-3-carboxylic acid	0.4118	-1.28	0.037748	1.4231
HMDB0000156	Malate	0.4256	-1.2324	0.04383	1.3582
HMDB0013286	N-Undecanoylglycine	0.43474	-1.2018	0.0001087	3.9636
HMDB0029653	1-(1-Methoxy-1-methylethyl)-4-methylbenzene	0.4372	-1.1936	0.045555	1.3415
HMDB0000543	Cumate	0.46245	-1.1126	0.0014671	2.8335
HMDB0029735	5-Ethynyl-5''-(1-propynyl)-2,2''-bithiophene	0.48866	-1.0331	2.54E-17	16.596
HMDB0003417	Cys	0.49635	-1.0106	2.88E-32	31.541
HMDB0000639	Galactaric acid	0.4973	-1.0078	2.16E-34	33.665

Supplementary table 4: Patient characteristics of the second validation cohort Related to Figure 4				
ID_Metabolomics	Disease	SEX	AGE	STAGE_NUM
ES_1	Melanoma_baseline	M	56	I
ES_2	Melanoma_baseline	M	69	I
ES_3	Melanoma_baseline	M	31	II
ES_4	Melanoma_baseline	M	71	II
ES_5	Melanoma_baseline	M	76	I
ES_6	Melanoma_baseline	M	83	II
ES_7	Melanoma_baseline	F	74	I
ES_8	Melanoma_baseline	F	63	II
ES_9	Melanoma_baseline	F	83	I
ES_10	Melanoma_baseline	F	56	II
ES_11	Melanoma_baseline	M	70	I
ES_12	Melanoma_baseline	M	67	I
ES_13	Melanoma_baseline	M	74	II
ES_14	Melanoma_baseline	M	35	I
ES_15	Melanoma_baseline	M	73	I
ES_16	Melanoma_baseline	F	46	II
ES_17	Melanoma_baseline	M	65	I
ES_18	Melanoma_baseline	M	50	II
ES_19	Melanoma_baseline	M	82	II
ES_20	Melanoma_baseline	F	32	II
ES_21	Melanoma_baseline	M	83	II
ES_22	Melanoma_baseline	F	85	II
ES_23	Melanoma_baseline	M	71	II
ES_24	Melanoma_baseline	F	56	II
ES_25	Melanoma_baseline	M	74	II
ES_26	Melanoma_baseline	M	83	I
ES_27	Melanoma_baseline	M	81	I
ES_28	Melanoma_baseline	M	58	II
ES_29	Melanoma_baseline	F	87	II
ES_30	Melanoma_baseline	M	55	I
ES_31	Melanoma_baseline	M	58	II
ES_32	Melanoma_baseline	F	62	II
ES_33	Melanoma_baseline	M	71	II
ES_34	Melanoma_baseline	M	74	II
ES_35	Melanoma_baseline	M	48	I
ES_36	Melanoma_baseline	F	81	I
ES_37	Melanoma_baseline	F	58	II
ES_38	Melanoma_baseline	F	64	II
ES_39	Melanoma_baseline	M	75	II
ES_40	Melanoma_baseline	M	85	II
ES_41	Melanoma_baseline	F	44	II
ES_42	Melanoma_baseline	M	59	II
ES_43	Melanoma_baseline	M	45	II
ES_44	Melanoma_baseline	M	64	II
ES_45	Melanoma_baseline	M	70	II
ES_46	Melanoma_baseline	F	67	II
ES_47	Melanoma_baseline	F	86	II
ES_48	Melanoma_baseline	M	75	II
ES_49	Melanoma_baseline	M	76	II

ES_50	Melanoma_baseline	F	90	II
ES_51	Melanoma_baseline	F	55	II
ES_52	Melanoma_baseline	M	72	II
ES_53	Melanoma_baseline	F	46	I
ES_54	Melanoma_baseline	F	58	I
ES_55	Melanoma_baseline	F	84	II
ES_56	Melanoma_baseline	F	52	II
ES_57	Melanoma_baseline	M	57	II
ES_58	Melanoma_baseline	F	31	I
ES_59	Melanoma_baseline	F	53	I
ES_60	Melanoma_baseline	M	74	II
ES_61	Melanoma_baseline	F	64	II
ES_62	Melanoma_baseline	M	81	II
ES_63	Melanoma_baseline	M	76	II
ES_64	Melanoma_baseline	F	77	I
ES_65	Melanoma_baseline	F	51	II
ES_66	Melanoma_baseline	F	79	II
ES_67	Melanoma_baseline	M	67	II
ES_68	Melanoma_baseline	M	86	II
ES_69	Melanoma_baseline	M	76	II
ES_70	Melanoma_baseline	F	47	II
ES_71	Melanoma_baseline	M	80	II
ES_72	Melanoma_baseline	M	70	II
ES_73	Melanoma_baseline	M	45	II
ES_74	Melanoma_baseline	M	55	II
ES_75	Melanoma_baseline	F	70	II
ES_76	Melanoma_baseline	M	63	II
ES_77	Melanoma_baseline	M	74	II
ES_78	Melanoma_baseline	M	61	II
ES_79	Melanoma_baseline	M	72	II
ES_80	Melanoma_baseline	F	55	II
ES_81	Melanoma_baseline	M	67	I
ES_82	Melanoma_baseline	M	61	II
ES_83	Melanoma_baseline	F	83	II
ES_84	Melanoma_baseline	F	81	II
ES_85	Melanoma_baseline	M	67	I
ES_86	Melanoma_baseline	M	62	II
ES_87	Melanoma_baseline	F	60	II
ES_88	Melanoma_baseline	M	68	II
ES_89	Melanoma_baseline	M	75	II
C_ES_1	Control	M	51	NA
C_ES_2	Control	M	55	NA
C_ES_3	Control	M	36	NA
C_ES_4	Control	M	40	NA
C_ES_5	Control	M	49	NA
C_ES_6	Control	M	67	NA
C_ES_7	Control	M	75	NA
C_ES_8	Control	M	34	NA
C_ES_9	Control	F	42	NA
C_ES_10	Control	F	32	NA
C_ES_11	Control	F	46	NA
C_ES_12	Control	F	25	NA
C_ES_13	Control	M	32	NA
C_ES_14	Control	F	44	NA
